# Modeling of the Reaction Mechanism of Enzymatic Radical C–C Coupling by Benzylsuccinate Synthase

**DOI:** 10.3390/ijms17040514

**Published:** 2016-04-07

**Authors:** Maciej Szaleniec, Johann Heider

**Affiliations:** 1 Jerzy Haber Institute of Catalysis and Surface Chemistry, Polish Academy of Sciences, Kraków 30-239, Poland; 2 Laboratory of Microbial Biochemistry, and LOEWE-Center for Synthetic Microbiology, Philipps-University of Marburg, Marburg 35043, Germany; heider@staff.uni-marburg.de

**Keywords:** benzylsuccinate synthase, anaerobic metabolism, DFT, kinetic isotope effect, toluene metabolism

## Abstract

Molecular modeling techniques and density functional theory calculations were performed to study the mechanism of enzymatic radical C–C coupling catalyzed by benzylsuccinate synthase (BSS). BSS has been identified as a glycyl radical enzyme that catalyzes the enantiospecific fumarate addition to toluene initiating its anaerobic metabolism in the denitrifying bacterium *Thauera aromatica*, and this reaction represents the general mechanism of toluene degradation in all known anaerobic degraders. In this work docking calculations, classical molecular dynamics (MD) simulations, and DFT+D2 cluster modeling was employed to address the following questions: (i) What mechanistic details of the BSS reaction yield the most probable molecular model? (ii) What is the molecular basis of enantiospecificity of BSS? (iii) Is the proposed mechanism consistent with experimental observations, such as an inversion of the stereochemistry of the benzylic protons, *syn* addition of toluene to fumarate, exclusive production of (*R*)-benzylsuccinate as a product and a kinetic isotope effect (KIE) ranging between 2 and 4? The quantum mechanics (QM) modeling confirms that the previously proposed hypothetical mechanism is the most probable among several variants considered, although C–H activation and not C–C coupling turns out to be the rate limiting step. The enantiospecificity of the enzyme seems to be enforced by a thermodynamic preference for binding of fumarate in the pro(*R*) orientation and reverse preference of benzyl radical attack on fumarate in pro(*S*) pathway which results with prohibitively high energy barrier of the radical quenching. Finally, the proposed mechanism agrees with most of the experimental observations, although the calculated intrinsic KIE from the model (6.5) is still higher than the experimentally observed values (4.0) which suggests that both C–H activation and radical quenching may jointly be involved in the kinetic control of the reaction.

## 1. Introduction

Although hydrocarbons have been believed for a long time to be persistent against microbial degradation in the absence of oxygen, since 1986 many bacteria have been shown to degrade these compounds anaerobically [1,2,3]. All of these microbes employ unusual enzymes to enable a number of various initial reactions with the highly inert hydrocarbon substrates, depending on the organism and the respective hydrocarbon. These include oxygen-independent hydroxylation [4,5,6], carboxylation [7,8], hydratation of multiple bonds [9], reverse methanogenesis [10], and most notably, fumarate addition reactions [11]. The first proof of fumarate addition initiating anaerobic hydrocarbon metabolism was presented for toluene degradation by the denitrifying bacterium *Thauera aromatica* [12], which generates enantiospecifically (*R*)-benzylsuccinate from fumarate and toluene. The reaction was soon confirmed to represent the general mechanism of toluene degradation in all known anaerobic degraders [13], and the enzyme catalyzing this unusual reaction was identified as a new glycyl radical enzyme, benzylsuccinate synthase (BSS) [14,15,16,17,18]. Very similar initiation reactions by fumarate additions catalyzed by closely related glycyl radical enzymes have since been described for anaerobic degradation of xylenes, ethylbenzene, *p*-cresol, 2-methylnaphthalene, *p*-cymene, and even alkanes, which seem to be added to fumarate at their subterminal methylene groups (reviewed in [19]). BSS and the growing number of additional fumarate-adding enzymes have become model cases for environmental processes in contaminated soils and deep anoxic subsediment habitats in recent years, and their isotopic preferences and conserved sequences serving as templates for molecular probes are currently employed as tools for monitoring these processes *in situ* [20,21,22,23].

**Figure 1:**
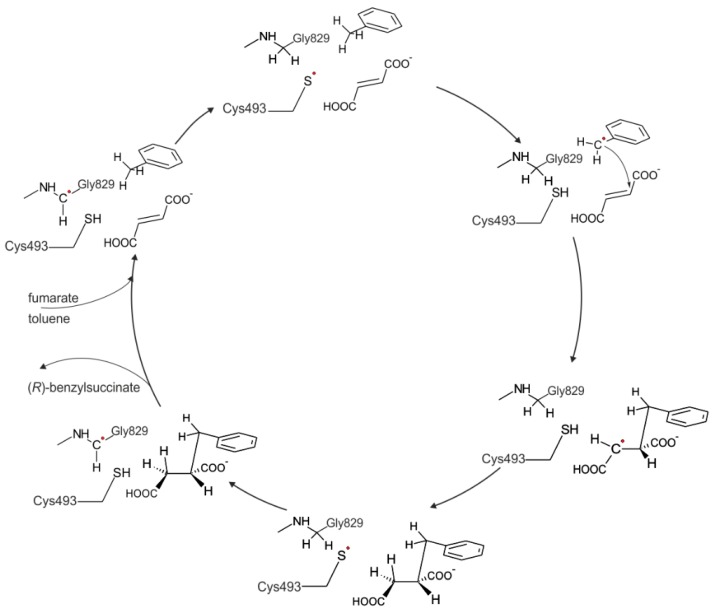
The postulated catalytic cycle for benzylsuccinate synthase (BSS).

All known BSS isoenzymes from anaerobic toluene degrading bacteria consist of three subunits of very different sizes: the large subunit of *circa* 100 kDa contains the glycyl radical in the active site and presumably carries out the catalysis, whereas the two small subunits of 8.5 and 6.5 kDa each contain a low-potential [4Fe4S]-cluster with still unknown function [13,14,24,25,26]. Like all glycyl radical enzymes, BSS needs to be post-translationally activated to the active, radical-containing state by a separate activating enzyme, which is encoded in a common operon with the genes for the BSS subunits and belongs to the family of *S*-adenosyl-methionine-dependent radical enzymes [13,27,28]. A hypothetical reaction mechanism of BSS has been proposed based on the general mechanisms of other glycyl radical enzymes, assuming that the glycyl radical present on a highly conserved glycine residue in activated BSS (Gly829) is initially transferred to an equally well conserved cysteine (Cys493). This generates a reactive thiyl radical in an active site cavity shielded from the environment, but containing the two substrates. The thiyl radical is then predicted to abstract a hydrogen atom from the methyl group of toluene, forming a benzyl radical as intermediate that would already be poised to stereospecific addition to the double bond of the fumarate co-substrate, yielding an (*R*)-benzylsuccinyl radical. This product radical will then be quenched by hydrogen transfer from the thiol group of Cys493, and after finally re-establishing the stable glycyl radical state of BSS by hydrogen transfer from Gly829 to the thiyl radical of Cys493, the product may be released and new substrates bound ([1], Figure 1). This mechanistic model was previously evaluated by gas-phase DFT modeling for activation of toluene and the aliphatic hydrocarbon butane and appears to be thermodynamically plausible [29,30]. Moreover, these studies suggested that the initial H-abstraction is the most probable rate limiting step and that radical quenching is also important to the overall kinetics [30].

Recently, the first reported crystal structure of a BSS in the non-activated state was presented [24]. Unfortunately, this structure did not contain any bound substrates or products, and therefore did not allow to formulate a more detailed mechanistic proposal. An earlier attempt to model the mechanism of BSS was based on a structural homology model of BSS derived from the structures of other GRE [31], which exhibited a number of significant deviations in the identity and conformation of predicted active site amino acids compared with the actual structure, especially regarding the crucial Arg508 residue in the active site. This amino acid is predicted to be a major player involved in substrate and product binding [24], but is not part of the active site in the structural model used, asking for some caution how to interpret the derived mechanistic data. In spite of that shortcoming, the enzyme-substrate complex proposed for this homology model was consistent with a *syn* addition of toluene to fumarate [31].

However, a number of mechanistic details that should be met by any theoretical model of BSS can be derived from the available enzyme-chemical data on BSS isoenzymes known from biochemical studies. Firstly, the reaction appears to be absolutely stereospecific, always leading to (*R*)-benzylsuccinate as only product [32,33]. Secondly, the abstracted hydrogen atom from the methyl group of toluene is donated back to the C3 atom of the benzylsuccinyl radical in a *syn*-addition mechanism [34], and thirdly, the stereochemistry of the hydrogen atoms of the methyl group of toluene appears to be inverted in forming the benzylsuccinyl product radical (unpublished data) [35], and the same stereochemical inversion has also been reported for an alkane-activating fumarate adding enzyme [36]. On the late stage of preparation of this manuscript two new BSS structures (PDB: 5BWE and 5BWD) were published by Funk *et al.* [37]. Both delivered the first experimental information on the enzyme with bound substrate(s). The authors co-crystalized fumarate with the enzyme and were able to show that it binds in a pro(*R*) manner with a decent resolution of 2.0 Å, exposing its double bond into the active site cavity. Unfortunately, the BSS complex with both fumarate and toluene in the active site yielded a significantly lower resolution of only 3.3 Å, resulting in significant uncertainties in positioning of the substrates, especially of toluene [37].

In this report, we present the first structure-based QM-model of the reaction mechanism involved in benzylsuccinate formation by BSS. We present the construction of several plausible models for the enzyme-product and enzyme-substrate complexes and present detailed calculations of four different mechanistic versions of the reaction, starting by radical activation of either toluene or fumarate and leading to the hypothetical production of either (*R*)- or (*S*)-benzylsuccinate. We also compare the outcome of our calculations with the recently published substrate-containing structures to assess the consistency of the modeled reaction mechanism.

### Possible Reaction Pathways

**Figure 2:**
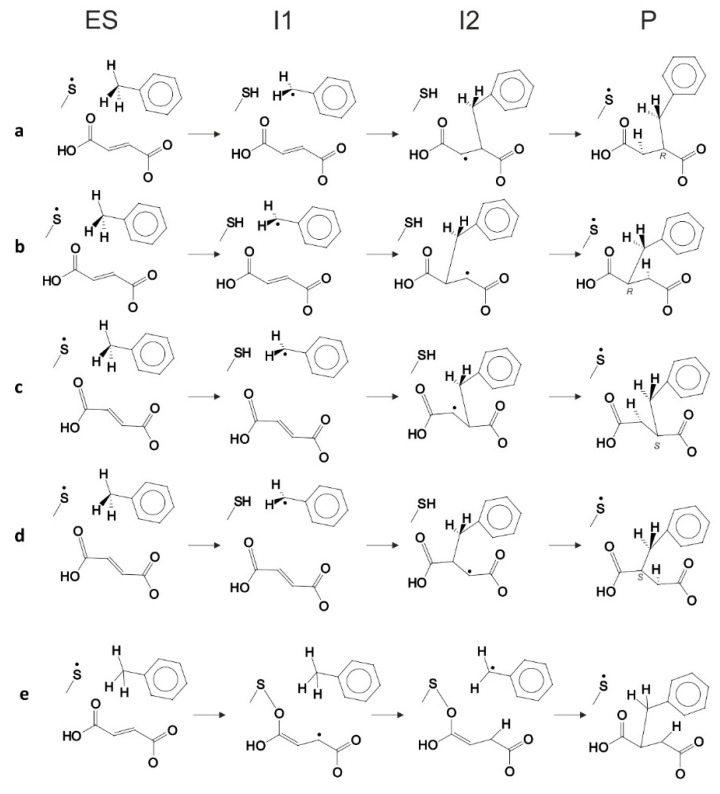
Four pathway variants considered in the study: pro(*R*)-oriented fumarate with the benzyl radical attacking (**a**) the distal C3 atom or (**b**) the proximal C2 atom of the fumarate; pro(*S*)-oriented fumarate with the benzyl radical attacking (**c**) the distal C3 atom or (**d**) the proximal C2 atom of the fumarate; (**e**) thiyl radical attacking carboxyl group of the fumarate.

In general, four variants of the catalytic pathway were analyzed (Figure 2). Pathways (**a**) and (**b**) lead to formation of (*R*)-benzylsuccinate and are initiated by hydrogen abstraction from toluene by the thiyl radical (**TS1**). The difference between pathways (**a**) and (**b**) consists of the subsequent attack of the benzyl radical at the carbon atoms of the double bond of fumarate, resulting in a new C–C bond. In variant (**a**), we assume that the distal atom (relative to Cys493) of the double bond (C3) is attacked (**TS2a**), whereas in variant (**b**) we assume the proximal (C2) carbon atom as target (**TS2b**). The quenching of the respective product radicals by Cys493 (**TS3a** and **TS3b**) leads to formation of (*R*)-benzylsuccinate in either variant.

The hypothetical pathway variants (**c**) and (**d**) were formulated to model the non-physiological formation of (*S*)-benzylsuccinate as alternative product, and they are otherwise identical to respective variants (**a**) and (**b**). Their crucial parts were studied, *i.e.*, toluene C–H activation (**TS1**), the attack of the benzyl radical on pro(*S*) oriented fumarate, either at the distal C3 (**TS2c**) or the proximal C2 (**TS2d**) carbon atom, formation of the respective product radical intermediates (**I2c** and **I2d**), their quenching by Cys493 (**TS3c** and **TS3d**) and formation of (*S*)-benzylsuccinate as product (**Pc** and **Pd**). The energy profiles of both pro(*R*) and pro(*S*) pathway variants were compared with respect to the pro(*R*)-ES complex.

Finally, the close proximity of the carboxyl group of the bound fumarate to the thiyl radical in the model (2.8–3.5 Å) prompted us to investigate additionally whether a radical attack of Cys493 on the oxygen atoms of fumarate may be feasible as an alternative initial reaction step that has never before been considered (pathway (**e**)). The structure of such specie was initially optimized without constraints, but after it proved to be unstable later, additional O–S bond constraints were added, followed by relaxed optimization.

## 2. Results

We initially attempted to obtain models of the enzyme-substrates complex by direct docking of fumarate and toluene into the crystal structure of the BSS α subunit. In order to achieve this, one has to dock one substrate first and the other later into the obtained complex. However, we obtained multiple possible docking poses of either fumarate or toluene, rendering it impossible to discern which of them would be catalytically active without additional information on the binding modes involved when both substrates are present. Moreover, even using a flexible docking protocol, the second substrate (e.g., toluene) was never successfully docked into any active site complex already containing the first substrate (e.g., fumarate).

**Figure 3:**
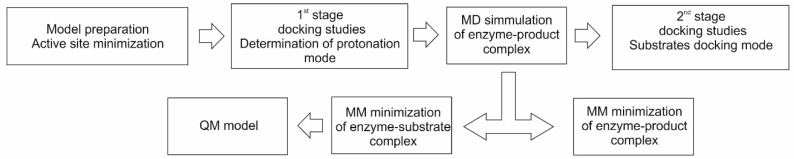
The modeling protocol used in the study.

Therefore to gain the required knowledge about the binding modes of the substrates, we tried an alternative approach, as suggested before in [31], by modeling the binding of the reaction product. The product (*R*)-benzylsuccinate is larger and much more conformationally constrained than the substrates fumarate or toluene, and as a result, consistent binding results were obtained. This allowed to determine possible protonation modes of the dicarboxylic acid part of the molecule and delivered an initial geometry of the enzyme-product complex for MD optimization, which finally also allowed to derive a model for the enzyme-substrate complex. The initial product-bound model was relaxed via MD simulations and used for a second stage of docking studies (Figure 3).

The knowledge obtained from the first stage of these docking studies allowed to select the catalytically active poses of the substrates toluene and fumarate out of the multiple results obtained previously from the docking protocol. Moreover, the MD simulation allowed the enzyme active site to attain a conformation suited to properly hosting both reagent molecules (and already accommodating the product to be formed).

### 2.1. Docking of the (R)- and (S)-Enantiomers of Benzylsuccinate (1st Stage)

In order to determine the energetically most favorable protonation state of the dicarbonic acid benzylsuccinate, all four possible protonation types of benzylsuccinate were docked into the active site. Moreover, to survey for any potential structural preferences for (*R*)-benzylsuccinate, the (*S*)-isomer was also docked into the active site (see the supplementary information for a detailed description). It should be underlined that docking and binding energy (BE) calculations are only a rough estimate of the influence of the enzyme on product protonation preferences, which was mainly used to discriminate predicted exergonic and endergonic binding modes.

All successful docking experiments revealed that the dicarbonic acid part of the benzylsuccinate is firmly positioned between the amide backbone group of Cys493 and the guanidino group of Arg508, which had already been predicted as important amino acids of the active site [24]. Analyzing the product binding energies obtained after geometry minimization of the active site of BSS (Table S1) revealed that the enzyme binds either completely protonated (*R*)-benzylsuccinate or its mono-protonated form facing Arg508 with the deprotonated carboxyl group (negative BE values). All binding poses presenting a charged carboxyl group towards Cys493 showed endergonic (positive) BE (from +14 up to +17.8 kcal/mol for the mono-protonated form, and +54.6 kcal/mol for the completely dissociated form). The best pose of the enzyme contained mono-protonated (*R*)-benzylsuccinate and was used in the following MD simulations.

Similar tendencies were observed for docking of (*S*)-benzylsuccinate. Although the best docking pose (mono-deprotonated oriented towards Arg508) showed the same calculated BE as for the (*R*)-isomer (−20.8 kcal/mol), we observed a higher variation on poses with that isomer, yielding more divergence of the calculated binding energies (from −20.8 to −9.14 kcal/mol), which might be due to a weaker accommodation of the product in (*S*)- than in (*R*)-configuration. However, the differences observed for the molecular interactions of BSS with the two product enantiomers were not clear enough to explain the enantioselectivity of BSS. Binding preferences with even more closely matching values between (*S*)- and (*R*)-benzylsuccinate have also been reported previously from Molecular Mechanics/Generalized Born Surface Area (MM/GBSA) calculations with the artificial active site predicted from the homology model [31].

### 2.2. Docking of the Substrates into the Relaxed Active Site

The structure of the BSS α subunit obtained in the initial MD simulation was used for more detailed docking studies of binding of the substrates (Table S2).

The substrate docking studies with fumarate were consistent with the previously presented preference of the enzyme to bind favorably the protonated or mono-deprotonated forms of the product. Exergonic binding was only predicted for mono-protonated fumarate facing Arg508 with the deprotonated group (BE −16.7 kcal/mol) or for fully protonated fumarate (−11.7 kcal/mol). In contrast, strongly endergonic BE were predicted for monoprotonated fumarate in the inverse orientation (+12.0 to +27.2 kcal/mol) or completely dissociated fumarate (+30 to +40 kcal/mol). The docking studies also suggest that the enzyme might be able to discriminate between binding of fumarate in either pro(*R*) and pro(*S*) orientation for the most favorable docking models of the mono-protonated forms. In this case, the fumarate binding in the pro(*R*)-oriented mode is more favorable than in pro(*S*)-orientation by *ca.* 5 kcal/mol, but this difference is already close to the error margin of the method [38].

### 2.3. Structure of the Enzyme-Product Complex

An MD simulation run with the α-subunit of BSS in complex with mono protonated (*R*)-benzylsuccinate yielded a relaxed structural model approximating BSS at the end of its expected reaction cycle, which contains the product-bound state of the active site with Cys493 in the thiol and Gly829 in the radical state. In order to speed up the calculations, the glycyl radical was not included into the model, because it is deeply buried in the interior of the enzyme and does not have access to the apparent substrate-binding cavity [24]. Therefore, we assume that its influence on the structure and reactivity of the active site is very limited. The last 1000 ps of the simulation were analyzed and the equilibrated structure with the lowest total energy was selected for further modeling. Although the original fold of the α subunit was preserved during the MD equilibration (Figure S1, root-mean-square deviation (RMSD) for backbone atoms 1.69 Å—Figure S2), a significant shift occurred within the active site residues (we recorded RMSD values of 1.56 and 2.02 Å for the main chain atoms and side chain atoms of the active site residues, respectively). The geometry of the equilibrated model was used as an initial point for further minimization of enzyme-substrate (ES) and enzyme-product (EP) complexes.

**Figure 4:**
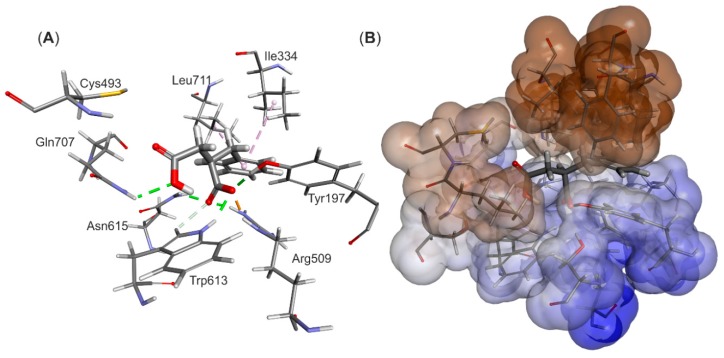
Structural model of the enzyme-product complex. (**A**) Binding mode of the (*R*)-benzylsuccinate (monoprotonated) and (**B**) EP complex depicting surface hydrophobicities of the active site residues (blue—hydrophilic, brown—hydrophobic). Interactions are shown by dashed lines in green (H-bond), orange (electrostatic interactions) and violet (vdW interactions).

The structural model of the EP complex model finally obtained (Figure 4) shows that the active site cavity contains two hydrophobic regions, which are involved in interacting with the benzene ring and the protonated carboxyl group, respectively. On the other hand, a hydrophilic portion of the active site cavity binds the deprotonated carboxylic group by a strong electrostatic interaction towards Arg508 and a range of hydrogen bonds. The analysis of interaction energies (IE) showed that the product is favorably stabilized in the active site (−36.6 kcal/mol) both by van der Waals (vdW) (−17.3 kcal/mol) and electrostatic (−19.3 kcal/mol) interactions. The strongest electrostatic and H-bond-based interactions are observed with Arg508 (−11.94 kcal/mol), but also Tyr197 (−3.16 kcal/mol), Trp613 (−2.52 kcal/mol), Gln707 (−0.85 kcal/mol) and Cys493 (−0.25 kcal/mol) are involved. The observed vdW interactions with the bound product are mainly formed with Ile384 (−2.39 kcal/mol), Leu492 (−2.34 kcal/mol), Leu391 (−1.45 kcal/mol), Val709 (−1.99 kcal/mol) and Leu711 (−1.90 kcal/mol) (see Table S1).

Surprisingly, similar enzyme-product complexes have been obtained after geometry minimization of the model with (*S*)-benzylsuccinate docked into the active site (Figure S3). The (*S*)-benzylsuccinate forms similar interactions in the enzyme active site: *i.e.*, a strong salt bridge with Arg508 (−11.35 kcal/mol), H-bonds with Tyr197 (−3.3 kcal/mol), Asn615 (−3.18 kcal/mol) and Cys493 backbone (−1.28 kcal/mol) as well as the hydrophobic contacts especially between phenyl ring and aliphatic amino acids (Leu384 and Leu711, −2.25 and −3.03 kcal/mol respectively). The overall strength of interactions with active site residues is a bit higher than that for (*R*) product (total interaction: −38.9 kcal/mol, vdW interaction −24.6 kcal/mol—see Table S3), and the (*S*)-benzylsuccinate forms significantly stronger electrostatic than vdW interactions compared to the (*R*) isomer. Although the analysis of interaction energy suggests that the (*S*)-isomer interacts stronger with the enzyme (especially with Arg508, Asn615 and Leu711) the analysis of BE suggests that (*R*)-benzylsuccinate is bound a bit tighter (by 7 kcal/mol) than the (*S*)-isomer (see Table S7). As the observed differences both in IE and BE are approximately 6%–10% of the calculated value and close to the method error it is not possible to univocally distinguish binding modes of both enantiomers. This results are also in line with previous analysis conducted for the homology model [31].

### 2.4. Structure of the Enzyme-Substrate Complex

**Figure 5:**
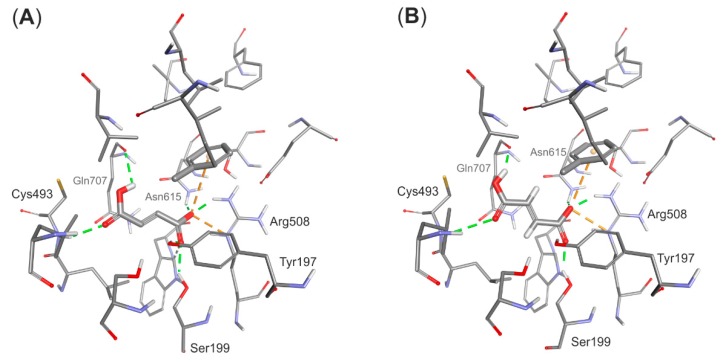
The structure of MM optimized enzyme-substrate complex for (**A**) pro(*R*)-oriented fumarate and (**B**) pro(*S*)-orientated fumarate. Green lines indicate H-bonds, orange lines ionic interactions. Amino acid that form H-bond interactions with fumarate were labelled.

Two principal models of enzyme-substrate complexes were studied: both contained a bound toluene, but one contained an additional pro(*R*)-, and the other a pro(*S*)-oriented fumarate (Figure 5). Both structural models were very similar (RMSD < 0.2) with the fumarate occupying approximately the same space in the active sites. The total energy of the pro(*S*)-oriented model was significantly higher than that of the pro(*R*)-oriented one (36 kcal/mol difference). The estimation of binding energies also confirmed that the pro(*R*) pose is more energetically favorable than the pro(*S*) equivalent (by 9.3 kcal/mol—Table S8).

Analysis of the interaction energies between fumarate and the rest of the active site residues plus the bound toluene revealed that the energetic advantage of fumarate binding in pro(*R*) orientation (−31.5 kcal/mol) amounts only to 1 kcal/mol (Tables S4 and S5). Fumarate is assumed to bind in mono-protonated form (charge −1), forming a strong electrostatic interaction with Arg508 (–10 kcal/mol) as well as H-bond interactions with Tyr197 (total interaction −3.4 kcal/mol), Ser199 (−2.1 kcal/mol), Cys493 (−3.1 kcal/mol), Trp613 (−1.36 kcal/mol), Asn615 (−2.2 kcal/mol) and Gln707 (−3.7 kcal/mol). Fumarate and toluene also favorably stabilize their mutual binding by interaction of the charged carboxylate group of fumarate with the pi electrons of the aromatic ring (about −2 kcal/mol).

The toluene is interacting with the active site (Table S6) mainly via vdW forces (by 98%) with a significantly smaller binding energy than that of fumarate: −19.7 kcal/mol for the complex in pro(*R*) and −19.2 kcal/mol for that in pro(*S*) orientation. The strongest interactions are those with fumarate (−2 kcal/mol, see above), and those with Ile384 (−2 kcal/mol), Arg508 (−2.2 kcal/mol) and Leu711 (−2.3 kcal/mol).

The structure of the BSS active site seems to be designed for tightly immobilizing the fumarate co-substrate, exposing its double bond towards the interior of the active site cavity. The toluene is simultaneously accommodated in a hydrophobic part of the active site, initially in a far distance from the potential radical sulfur (5–6.5 Å, depending on the conformation of Cys493—see Figure S4). On the other hand, the non-dissociated carboxyl group of fumarate is positioned in a rather close vicinity to the radical S atom (3.5–2.5 Å). Therefore, we regarded it as important to take novel mechanistic variants into consideration in which the thiyl radical of BSS may initially attack the fumarate, leading to the formation of potential fumaryl-based radicals (Figure 2, mechanism **e**).

**Figure 6:**
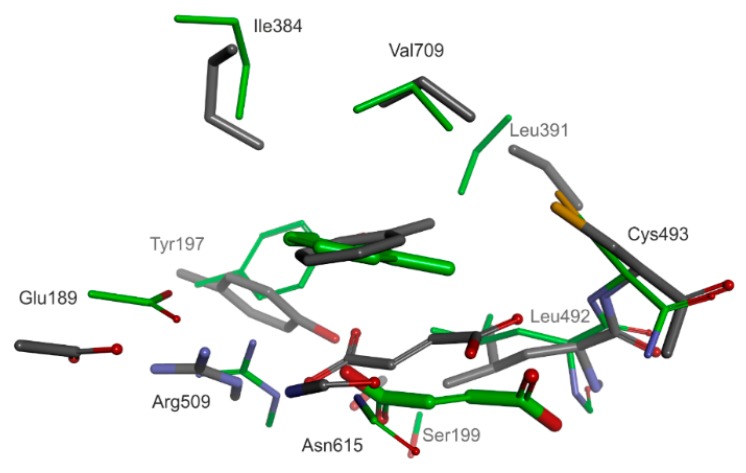
Comparison of the bound substrates in BSS as predicted in our model (**grey**) and a recent crystallographic study (**green**).

In order to compare the modelled ES complex with the recently published crystal structure of BSS we used a constrained cluster model where toluene was moved closer to the S atom of Cys493 (from 7.1 to 3.5 Å; see below for justification). The comparison revealed that our prediction comes very close to the observed structure (5BWE). The overlay of all heavy atoms of the cluster model and respective heavy atoms of the 5BWE active site revealed a significant concordance of the two structures (overall similarity for corresponding heavy atoms of 70%). The carboxyl group of fumarate facing Arg508 is basically poised at the same site in the model as observed in the structure, while the rest of the molecule is slightly tilted at an angle of approximately 28° (Figure 6). Moreover, the toluene also occupies about the same space in the model as in the structure (with a relative tilt of the aromatic ring of 12°). Both geometries of bound toluene would be consistent with the rather weakly resolved electron density attributed to this molecule in the structure [37]. It is also remarkable that both models predict the same mutual parallel orientation and relative distance of bound fumarate and toluene (3.6 Å distance between methyl group and C atoms of the fumarate double bond), which is a prerequisite for the reaction mechanism (Figure 6).

### 2.5. Reaction Pathway

The reaction pathways were studied with DFT using the cluster model presented in Figure 11. The benzyl-radical dependent pathway variants studied here (**a**–**d**) are described by the following abbreviations for consistent labelling of the respective stationary points: **ES** represents the enzyme substrate complex, **TS1** the transition state of the toluene C–H activation reaction by the Cys493 radical, and **I1** corresponds to the benzyl radical intermediate and fumarate present in the active site. The subsequent transition state involved in C–C bond formation is described by **TS2** for the attacks on C3 (variants **a** and **c**) or C2 (variants **b** and **d**) of the fumarate, leading to state **I2** containing the benzylsuccinyl radical intermediate. Finally, **TS3** represents the third transition state for the quenching of the benzylsuccinyl radical by a hydrogen transfer from the Cys493-SH group, leading to the enzyme-product complex **P** with bound benzylsuccinate. The added labels **a**–**d** identify the association of the respective mechanistic states with the proposed pathway variants.

#### 2.5.1. Pro(*R*)-Specific Pathways

**Figure 7:**
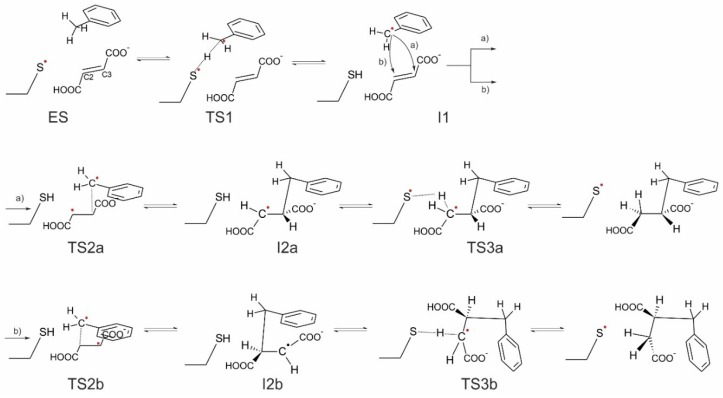
The mechanism of reaction with two variants: attack on distal carbon atom (pathway (**a**)) of the double bond of fumarate and attack on the proximal carbon atom (pathway (**b**)).

**Figure 8:**
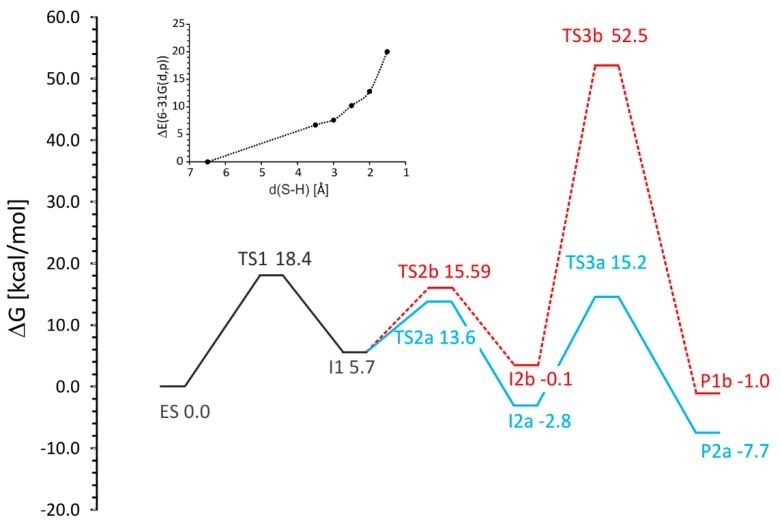
The Gibbs free energy profile obtained for pro(*R*) pathway with two variants (**a**)—solid blue line and (**b**)—dashed red line. The solid black line depicts a common part for both variants (*i.e.*, ES-I1). The small panel depicts the distance dependence of electronic energy values from scanning intermediates of a gradual approach of bound toluene towards the Cys493 radical before reaching the enthalpy value of TS1.

The generally accepted mechanistic proposals on glycyl radical enzymes predict the reaction to start with an H atom transfer from Cys-SH to the Gly radical. This step is supposed to be triggered by the close spatial proximity of the two residues and can be considered as highly probable in BSS, based on a distance of only 2.6 Å between Gly829 and Cys493 in the structure [24]. Because this initial step occurs outside the apparent active site cavity and is not directly involved in the reaction of the substrates, we decided to omit it in our modeling process. Therefore, our model of the reaction pathway starts with Cys493 already in the thiyl radical state and activating the bound toluene (or fumarate) in the active site (Figure 7). In order to achieve hydrogen abstraction from toluene, the methyl group has to approach the Cys-S atom to a distance of about 3 Å d(S–C). However, our cluster model predicted a longer distance of 6.5 Å between the sulfur of the thiyl radical and the H of the methyl group of bound toluene. To evaluate the significance of this fact, we scanned the potential energy values of several intermediate states during the approach of toluene towards the thiyl radical from a S–H distance of 6.5 to 1.5 Å. We found a relatively low energy increase of the model during the initial phase up to a distance of 3 Å (amounting to *ca.* 35% of the **TS1** energy barrier—see Figure 8), while a much sharper rise occurs at S–H distances of less than 2.5 Å (equals to an S–C distance of 3.38 Å). The best resemblance of the model with the crystal structure was reached at a d(S–C) distance of 3.58 Å (Figure 6) and the thermally corrected energy of this model exhibited a 3.5 kcal/mol higher energy than the reference ES structure.

The modelled transition state (**TS1**—see Figure S5) for the hydrogen atom transfer reaction from toluene to the thiyl radical exhibits almost equal C–H and S–H bond distances (1.49 and 1.51 Å, respectively) and an S–H–C angle of 163.5°, which is close to the optimum value of 180° for exchanging the H radical. Two thirds of the spin density are located on the toluene (54% thereof localized on the methyl carbon), while 33% are still localized on the S atom of Cys493. The activation leads to the formation of a benzyl radical (Figure S6), which is again accommodated in a hydrophobic pocket in close vicinity of the fumarate double bond (distances of 3.6 and 3.9 Å to the C2 and C3 atoms of fumarate, respectively). At this point, the benzyl radical can undergo two possible reactions (Figure 7)—to attack at either the proximal or distal (with respect to Cys493) carbon atom of the fumarate double bond. As both carbon atoms are in a close vicinity to the benzyl radical and the proximal C2 carbon atom is even closer (3.6 Å), we have decided to investigate both possibilities. Pathway (**a**) implies the attack on the distal carbon atom (C3), pathway (**b**) on the proximal one (C2).

##### Pathway (a)

The mechanistic variant of forming a C–C bond between the benzyl radical and the distal C3 carbon atom of the double bond of fumarate proceeds with *syn* configuration in the product. This variant also leads to a stereochemical inversion of the H-atom configuration at the methyl group of toluene in the benzylsuccinyl radical intermediate (Figure S7). In the correlated transition state (**TS2a**) the C–C bond distance between the methyl group of the benzyl radical and C3 of fumarate is 2.25 Å and most of the spin density (0.71) is still located on the benzyl radical with the rest localized on fumarate (0.29), mainly on the proximal (C2 0.36) and distal (C3 −0.16) carbon atoms of fumarate. The spin of the product radical intermediate (**I2a**—Figure S8) is mainly localized on the proximal (C2) carbon atom (0.88) and only a fraction of it is still localized in the aromatic ring (0.11). The reaction is finished by a back-transfer reaction of the hydrogen atom from Cys493 to the product radical. The correlated transition state (**TS3a**) exhibits again very short S–H and C–H distances of 1.58 and 1.50 Å and an S–H–C angle of 174° (Figure S9). Most of the spin density in **TS3a** is still localized on the benzylsuccinyl radical (0.63) with only 0.37 already transferred to the S atom. It should be underlined that this quenching process of the **I2a** radical is associated with severing only one H-bond between the carboxyl group of fumarate and the amide bond of Cys493, while most other intermolecular contacts remain intact. Completion of the H transfer results with the formation of the product in (*R*) configuration (Figure S10).

##### Pathway (b)

The alternative mechanistic variant of forming the C–C bond between the benzyl radical and the proximal (C2) carbon atom of the fumarate leads to a very similar transition state (**TS2b**) with a C–C bond length of 2.23 Å. Again, most of the spin density is localized on benzyl radical (0.66). The overall spin of fumarate (0.33) is mostly distributed over the C3 (0.5) and C2 atoms (−0.19). After completion of C–C bond formation, a benzylsuccinyl radical intermediate is formed (**I2b**) which is associated with stereochemical inversion of the H-atom configuration at the benzylic carbon atom of toluene as in pathway (**a**). In **I2b**, the radical spin is mostly localized on the distal carbon atom of the former fumarate, which is oriented towards Arg508 via the adjacent deprotonated carboxyl group. As a result, the radical of the benzylsuccinyl intermediate can only be quenched after braking the contacts of the deprotonated carboxyl group within the binding site and rotating the intermediate to position the distal carbon atom closely enough to the Cys-SH group. In **TS3b**, the predicted S–H and C–H bond lengths are 1.62 and 1.55 Å, respectively, and the correlated S–H–C angle of 143° is significantly less linear (and therefore unbefitting to the required hydrogen transfer) than in **TS3a**, while a similar spin distribution is predicted (0.33 on the S atom, 0.7 on the product radical). Moreover, restoration of the abstracted hydrogen is only possible in an anti-conformation, which contradicts the experimental evidence. After completion of the reaction in this mechanistic variant, (*R*)-benzylsuccinate would be re-instated into the former configuration, re-establishing the contacts of the carboxyl group with Arg508 (**Pb**).

##### Energy Profiles

The reaction energy profiles presented in this study were calculated with a range of corrections to the electronic energy. All values are available in the Table 1 and in the supplement (Table S9) which allows evaluation of the effects introduced by particular corrections. As the Gibbs free energy values deliver the most complete description of the studied system, they were mainly used for our evaluations as described in the text (*i.e.*, B3LYP+D2/6-311+g(2d,2p)+G^corr^+ solvent correction calculated on B3LYP+D2/6-31g(d,p) level).

The analysis of the energy profiles (Table 1, Figure 8) obtained for variants (**a**) and (**b**) shows the following observations:

- Toluene C–H activation (**TS1**) is predicted to be the highest energetically demanding step of the reaction (18.4 kcal/mol)
- C–C– bond formation shows an energetic preference for attacking the distal (**TS2a**, 13.6 kcal/mol) over the proximal C atom of the double bond (**TS2b**, 15.6 kcal/mol)
- The introduction of entropy effect into potential energy profile results with increased energy of **I2a** by 5 kcal/mol. As a result the **TS1** is a rate limiting step compared to **TS3a**. (Δ*G* of 18.4 *vs.* 18.0 kcal/mol). For energy representation without entropy corrections (electronic energy, *E* + ZPE or *E* + thermal energy) the **TS3a** barrier (with respect to **I2a**) is higher than that calculated for **TS1**.
- **TS3b** requires severing of the salt bridge interaction with Arg508 and therefore is associated with a prohibitively high energy barrier of 52.5 kcal/mol
- The overall reaction is exergonic (−7.7 kcal/mol)

The above analysis allows to exclude pathway **b** due to a slightly higher barrier of the C–C bond formation (**TS2**) and especially the prohibitively large energy barrier associated with the radical quenching step (**TS3b**). Assuming that intermediate **I2b** would be formed, the height of the **TS3** energy barrier would not allow further reaction progress and the intermediate would decompose to **I1**.

#### 2.5.2. Pro(*R*) *vs.* Pro(*S*)-Oriented Pathways (Pathway (**a**) *vs.* Pathway (**c**) and (**d**))

BSS is reported to be enantioselective in exclusively forming (*R*)-benzylsuccinate. To elucidate potential factors enforcing this enantioselectivity, we have analyzed hypothetical pathway variants leading to the (*S*) product. We started these studies with geometry optimization of the cluster model with the fumarate oriented in a pro(*S*) manner. As expected from our previous calculations, the fumarate occupies the same space as in the model exhibiting pro(*R*) orientation and also forms a salt bridge with Arg508 and H-bonds with Tyr197, Asn615 and the backbone amide group of Cys493. The analysis of the energy of this model showed a slightly higher value for the **ES** complex than for the corresponding complex in pro(*R*) orientation (difference of 3.1 kcal/mol for ZPE corrected profile, 4.0 kcal/mol for ∆*G*) (Table 2 and Table S10). This confirms the results from the modeling of ES complexes by MM modeling which indicated that formation of the pro(*S*)-ES complex requires additional energy and is less thermodynamically probable.

Although we were not able to obtain a stable value for **TS1** for pathway **c** due to periodic displacements of the Leu492 side chain, the transition state was fairly well approximated yielding ∆*G*^#^ of 16.93 kcal/mol, *i.e.*, a slightly lower value (by 2.5 kcal/mol) than observed for pro(*R*) pathway.

The calculated ∆*G*^#^ of the transition state for C–C bond formation for the distal C3 carbon atom (**TS2c**) is higher (15.9 kcal/mol compared to 13.6 kcal/mol for pro(*R*)), while the principal geometric features stay very similar (C–C bond distance of 2.23 Å). On the other hand the C–C bond formation for the proximal C3 carbon atom (**TS2d**) is associated with energy barrier (13.4 kcal/mol) which is lower than that of **TS2c** by 2.5 kcal/mol and amounts to the same energy as in case of **TS2a**.

The benzylsuccinyl radical-bound state **I2c** shows a slightly lower Gibbs free energy than in case of **I2a,** but the subsequent step to **TS3c** involved in radical quenching exhibits a significant higher ∆*G*^#^ of 22.0 kcal/mol (with respect to **I2c**), despite the very similar values of the S–H and C–H distances (1.55 Å) between variants (**a**) and (**c**). Meanwhile the intermediate state **I2d** exhibits an almost identical energy to that of **I2a** (−2.7 kcal/mol), but is followed by a prohibitively high barrier to **TS3d** (34.4 kcal/mol) associated with radical quenching by Cys493.

The overall energetics of the hypothetical reactions leading to the formation of (*S*)-benzylsuccinate are very similar (−9.1 *vs.* −5.4 kcal/mol for **Pc** and **Pd**).

#### 2.5.3. Alternative Fumaryl-Radical Dependent Mechanistic Variants

To cover the full spectrum of alternative mechanistic variants of the BSS reaction, we also evaluated a fundamentally different alternative mechanistic hypothesis assuming an initial attack of the Cys-S-radical at the fumarate co-substrate. A hypothetical attack of the thiyl radical at the adjacent carboxyl group of fumarate may lead to a covalently bridged cysteinyl-fumaryl radical species that may subsequently abstract a hydrogen atom from toluene and lead to radical addition of the benzyl radical to the re-formed fumarate (variant **e**). However, our calculations discarded this mechanistic variant because of the instability of the predicted cysteinyl-fumaryl radical species. This radical intermediate is unstable and therefore must be expected to decompose immediately to fumarate and the Cys-radical (see Figure S11). Therefore, it seems that the carboxyl group of fumarate, despite close proximity to thiyl radical, is not prone to accidental activation.

A second hypothetical possibility of a fumaryl-radical based mechanistic variant may be an attack of the thiyl group at the double bond of fumarate, yielding a thioether-bonded covalent cysteinyl-fumaryl radical intermediate. However, this mode of initial reaction was also discarded due to the long distance (more than 5 Å) between the thiyl radical and the proximal C atom of the double bond. This reaction would require extensive reorientation of the tightly bound fumarate accompanied with a prohibitively large energy barrier (see above).

### 2.6. The Kinetic Isotope Effect

The BSS reaction mechanism was probed by kinetic studies using isotope-labelled substrates. BSS exhibits a strong kinetic isotope effect, as evident from comparing the specific activities measured with [^2^H]_8_-toluene and unlabeled toluene. Extracts of toluene-grown *T. aromatica* cells yielded values of 4.0 *vs.* 16 nmol·min^−1^ (mg·protein)^−1^, indicating a fourfold slower turnover rate of the deuterated substrate (Seyhan *et al.* unpublished) [35]. In contrast, assays with deuterated fumarate (2,3-[^2^H]_2_-fumarate) yielded the same turnover rates as those with unlabeled fumarate. Therefore, we assume a kinetic isotope effect of 4.0 for [^2^H] substituents at the methyl group of toluene. This is in contrast with the somewhat lower KIE values of 1.7 ± 0.2 reported earlier using a different strain of *T. aromatica* [39].

In order to validate our theoretical model, we have calculated the intrinsic KIE associated with all transition states taking the **ES** complex as a reference (see Table S11). For each of the steps the KIE was calculated based on Gibbs free energy corrections. The calculated predicted KIEs are a combination of primary (due to H/D transfer) and secondary (due to the presence of deuterons in adjacent bonds) kinetic isotopic effects. The calculations show that both **TS1** and **TS3** are associated with similar, kinetically indistinguishable KIE values of 6.9 and 6.7, respectively. **TS2** is predicted to be associated with a strong secondary KIE of 2.0. As the **I2** intermediate is characterized by the lower free energy than ES and the overall barrier (**TS3** *vs.* **I2**) is very close to that calculated with respect to **ES** we have also calculated the respective KIE taking **I2** as a reference. In such case the intrinsic KIE associated with **TS3** step would be 4.0.

## 3. Discussion

### 3.1. BSS Enzyme-Substrate Model

In this communication we present the first structure-based model calculation on the reaction mechanism of the radical-based addition of fumarate to toluene by benzylsuccinate synthase. The beginning of this study preceded the publication of the structure of the ES complex of BSS (5BWE) and therefore it was not possible at that time to construct a plausible model of the enzyme-substrate complex directly. Nevertheless, we were able to obtain a model of a complex with the bound product. Using this enzyme-product complex as starting point, consistent models of BSS containing both bound substrates were obtained and used to identify important active site amino acids. Binding of the substrates and the product seems to be dominated by electrostatic and H-bonding interactions with the distal carboxyl groups (relative to the active-site Cys493) of either the product benzylsuccinate or the co-substrate fumarate, which apparently need to be in the fully protonated or mono-deprotonated state. The proximal carboxyl groups of the bound product or of bound fumarate are predicted to be in the non-charged protonated state and bound via backbone contacts to Cys493 and vdW interactions, and the aromatic rings of either benzylsuccinate or the substrate toluene are also coordinated via vdW interactions. The predicted structure of the model with bound fumarate and toluene turned out to be in good agreement with the crystal structure published by Funk *et al.* [37], which shows only moderate resolution, especially for the position of bound toluene. The main difference in our model is a closer proximity of fumarate to Cys493 and a longer distance between the methyl group of toluene and the S-atom of Cys493 for the ES complex. Despite these differences, the positioning of the reagents in TS1 of our cluster model can be easily superimposed on active site residues of the structural model (5BWE), indicating that all important steric and electrostatic interactions are accounted for in our modeling and the predictions on the reaction mechanism are valid.

The obtained models of enzyme-substrate and enzyme-product complexes were used to select the active site amino acid side chains to be considered for QM modeling of the course of different hypothetical mechanistic variants of the reaction pathway. We analyzed two versions of the generally favored mechanism initiated by formation of a benzyl radical from toluene, a variant leading to the non-physiological enantiomer (*S*)-benzylsuccinate, and a mechanism initiated by formation of a fumaryl radical for their thermodynamic and mechanistic plausibilities.

### 3.2. Mechanistic Variants Initiated by Benzyl-Radical Formation

**Figure 9:**
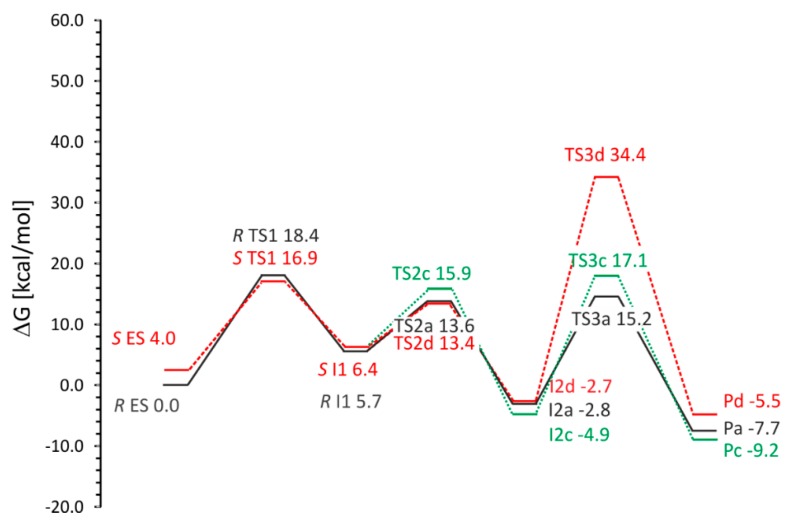
The Gibbs free energy profile obtained for pro(*R*) pathway (a)—solid black line and pro(*S*) pathways: (c)—dotted green line and (d)—dashed red line.

**Figure 10:**
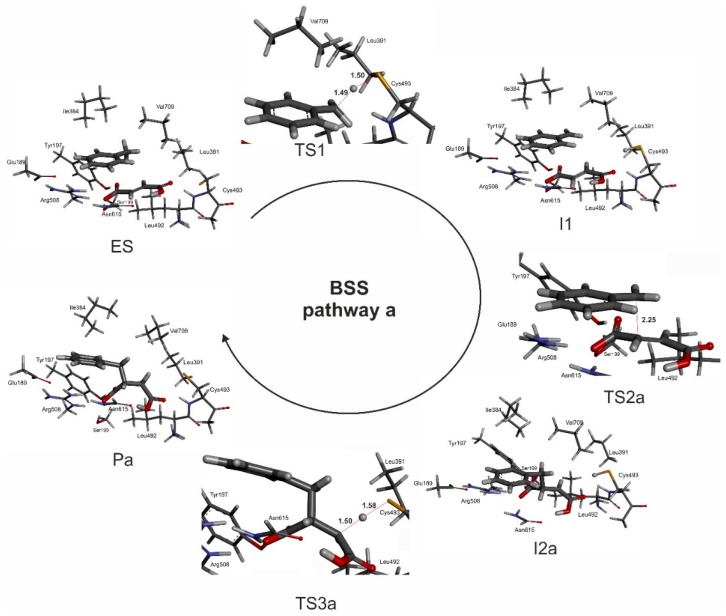
The reaction catalyzed by BSS (variant **a**).

We decided to omit the predicted initial radical transfer from the glycyl radical to Cys493 in our study and to start the calculations with a model of the enzyme-substrate complex already containing the reactive thiyl radical at Cys493, because Gly829 is localized in the hydrophobic interior of the BSS structure and does not have access to the substrate-binding cavity [24]. The first mechanistic variants to be considered corresponded to those used in all previous proposals and theoretical models on the BSS reaction [1,29,31] in being initiated by transferring a hydrogen atom from the methyl group of toluene to the thiyl radical of Cys493 (Figure 9). However, the predicted enzyme-substrate complex exhibited a rather large distance between the thiyl radical and the methyl group of toluene (6.5 Å) which initially appeared to disagree with the postulated hydrogen transfer. This contradiction has been solved during the mechanistic calculations, which predict an initial approximation phase of the toluene towards the thiyl radical with a rather shallow increase of energy, while the main contributions of transition state formation occur only after the distance is shortened to less than 3 Å. The overall reaction was arranged into three sub-steps (Figure 10), consisting of hydrogen transfer from toluene to the thiyl radical of Cys493, C–C bond formation between the benzyl radical and fumarate, and a final hydrogen transfer from Cys493 to the benzylsuccinyl product radical, yielding a mechanistic route from the enzyme-substrate complex (**ES**) to the enzyme-product complex (**P**) via three transition states (**TS1**-**3**) and two intermediate states (**I1** and **I2**). The main model (variant **a**) predicts formation of the C–C bond between the benzyl radical and the distal C-atom (C3, relative to Cys493) of the double bond of fumarate. It showed reasonable energy values of all three transition states, predicting the hydrogen abstraction from toluene (**TS1**) as most probable rate-limiting step (followed and possibly rivalled only by the final step of quenching the product radical and reconstituting the thiyl radical). This result is consistent with recently conducted gas-phase calculations for isolated reagents [30]. Moreover, the calculated model is enantiospecific in yielding (*R*)-benzylsuccinate and predicts correctly the expected stereochemical inversion of the methyl group of toluene and the *syn*-addition of the reconstituted hydrogen to the product radical.

### 3.3. Selection of the C Atom Added to the Benzyl Radical

In order to identify which of the C-atoms of the double bond of fumarate is actually involved in forming the new C–C bond of benzylsuccinate, we calculated the energetic courses of both mechanistic variants, where the benzyl radical attacks either the distal (C3) or the proximal (C2) atom of the fumarate co-substrate. The results allowed a clear discrimination between the two possibilities and eliminated any possible radical addition at C2 of fumarate, because the resulting product radical cannot reasonably be reconstituted to the product. The required back-transfer of the abstracted hydrogen from Cys493 to the C3-atom of the product radical would require unreasonable amounts of reorganization energy and additionally lead to an *anti*-addition phenotype incompatible with the known properties of BSS.

### 3.4. Stereospecificity

To rationalize the factors mediating the observed stereospecifity of BSS in exclusively producing (*R*)-benzylsuccinate, we calculated the course of a hypothetical reaction under conditions producing the non-physiological (*S*)-enantiomer. To our surprise, only minor differences were predicted for binding of either (*S*)-benzylsuccinate (BE 7 kcal/mol higher than for pro(*R*)) or the enzyme-substrate complex with fumarate in pro(*S*)-orientation (9 kcal/mol), compared to the data obtained with the physiologically oriented complexes. The energetic preference for the *R* orientation of substrates seems to be caused by conformational energy differences in the enzyme-substrate complexes which may contribute to the enantiospecificity of the reaction (36 kcal/mol difference between pro(*S*) and pro(*R*) complexes). A similar result was obtained for a QM-only cluster model where the pro(*S*) enzyme substrate complex was characterized by a 4 kcal/mol higher free energy than for pro(*R*) pathway.

A second possible contribution to explain the enantiospecificity of BSS came from our investigation of predicted free energy reaction profiles. Binding of fumarate in the pro(*S*) manner results with the reversed preference of the attack of the benzyl radical on the carbon atom of the fumarate double bond. As a result the benzyl radical would preferentially attack the proximal C2 atom in the pro(*S*) pathway, which leads to a prohibitively high barrier of radical quenching process (**TS3** 34.4 kcal/mol).

Therefore, it seems that a combination of thermodynamic (lower probability of pro(*S*) **ES** formation) and kinetic factors (preferential pathway leads to prohibitive **TS3** in case of pro(*S*) **ES**) is responsible for the enantiospecificity of the enzyme.

### 3.5. Mechanisms Initiated by Fumaryl-Radical Formation

The predicted structure of the enzyme-substrate complex of BSS showed that one of the carboxyl groups of fumarate is located much closer to the thiyl radical group of Cys493 than the methyl group of toluene. This prompted us to determine the plausibility of an initial formation of a fumaryl radical species as depicted in Figure S11. One may predict the formation of a covalent adduct of the thiyl sulfur to the carboxyl group of fumarate, which would produce a transient covalent cysteinyl-fumaryl adduct extending the radical function towards the bound toluene. The fumaryl radical may then abstract a hydrogen from toluene, generating a benzyl radical that adds to the double bond of either the fumaryl adduct or the restored fumarate (Figure 2d). After calculating the initial steps of this alternative hypothesis, it became clear that the predicted cysteinyl-fumaryl radical species is thermodynamically too unstable to play a role in the BSS mechanism, eliminating this type of reaction initiation. An alternative pathway of forming a covalent fumaryl radical species by attacking directly at the double bond was eliminated because of the large distance between the thiyl radical and C2 of the bound fumarate.

### 3.6. Predicted KIE

The predicted intrinsic KIE is larger than the observed KIE determined experimentally. However, the barriers calculated here for toluene and its labelled isomer (see Table S11) are a good starting point for a more thorough kinetic modeling of the BSS reaction mechanism. The lower value of the observed KIE suggests that the reaction kinetics may be controlled not only by toluene activation (**TS1**) but also further steps that are not associated with such a high intrinsic KIE. It is possible that in a real, non-static, moving enzyme both **TS1** and **TS2** barriers are much closer to each other and both may influence the kinetics of the reaction or the observed kinetics is under control of both **TS1** with reference to **ES** and **TS3** but with reference energy of **I2**. Finally, it should be underlined that the difference in calculated KIE (6.9) and experimental value (4.0) corresponds to a very small energy difference between both barriers (0.3 kcal/mol) which is well within the error range of the DFT calculation and the cluster calculation represents only a single geometry of the enzyme active site, which is just one representation of many possible conformations of the active site. A sensitivity analysis of simulated reaction rates by Bharadwaj *et al.* [30] has already indicated that both processes, *i.e.*, C–H activation (TS1) and radical quenching (TS3), are most significant for the overall kinetics, and variations in their barriers will strongly influence the overall reaction rate.

## 4. Experimental Section

### 4.1. Initial Model Preparation

The initial geometry of the α subunit of BSS was taken from the solved crystal structure of the enzyme from *Thauera aromatica* strain T1 ([24]; PDB code: 4PKF, resolution 2 Å). The β and γ subunits as well as water molecules were omitted, and protons were added by the “Calculate Protein Ionization and Residue pK” protocol of Biovia Discovery Studio (DS) 4.0, assuming an optimum pH value of 7.4. All calculations in DS 4.0 were conducted using a CHARMm force field [40,41]. After local geometry minimization of the active site residues (all residues in 5 Å radius from Cys493), the obtained model was used for docking of fumarate, toluene or benzylsuccinate, using all four possible protonation states for the acids (fully protonated acids without charge, single deprotonation at either carboxyl group with charges −1, and double deprotonation with charge −2) by the LibDock protocol (flexible docking with *in situ* energy minimization; parameters of the docking protocol are available in the Supplement) [42]. The obtained poses were evaluated by calculation of the binding energies using a Distance-Dependent Dielectrics model solvent [43]. The best-fitting protonation state of the product-bound state (charge −1 with the deprotonated group pointing toward Arg508) was selected based on the lowest binding energy (BE) of the respective complex. BE was calculated as a difference between energy of the enzyme-ligand complex (Complex Energy) and sum of energies of the enzyme (Protein Energy—same conformation but without ligand) and free ligand (Ligand Energy) [38]. A similar approach was used for analysis of the recently published BSS-fumarate complex (5BWE). The fumarate position in the alpha subunit was minimized and the model was subjected to the LibDock protocol. Furthermore, the whole geometry of the alpha subunit was minimized in Generalized Born with Molecular Volume correction (GBMV) [44] model solvent (see modeling of the enzyme substrate complex for details) and the docking was repeated.

### 4.2. Molecular Dynamics

The obtained model of an enzyme-product complex was solvated by 14187 water molecules (TIP3P) in a periodic boundaries box (83.7 Å × 73.2 Å × 97.7 Å), and sodium and chloride ions were added to maintain a neutral charge and constant ion strength of the system (ion concentration 0.145 M). All calculations were conducted using Discovery Studio 4.0 (Biovia) using CHARMm force field [40,41]. The initial geometry was minimized with the steepest descent algorithm (until RMS gradient of 0.01 kcal·mol^−1^·Å^−1^) followed by a conjugate gradient (RMS gradient cutoff at 0.00001 kcal·mol^−1^·Å^−1^). The minimized geometry was subjected to an MD simulation cascade consisting of *in-silico*-“heating” up to 300 K for 10 ps, a temperature equilibration phase for 1000 ps and a simulation phase for 5000 ps conducted in the NPT system, with 2 fs time step and using SHAKE constraint for fixing all bonds involving hydrogens in the simulation. The obtained trajectory of the simulation phase was analyzed and the frame with the lowest total energy was used as a model for further docking and molecular mechanics studies.

### 4.3. Modeling of the Enzyme Substrate Complex

The bound product molecule was replaced by the substrates toluene and mono-protonated fumarate, using the product coordinates to obtain a starting geometry for the reactants. The side chain of Cys493 was modified to represent a thiyl radical state, and its point charges were calculated by DFT (B3LYP/6-31g(d,p), IEFPCM, ε = 4) according to the Merz-Singh-Kollman method [45,46] and the CHARMm SE type (thioether sulfur) was used to describe the rest of the radical S parameters. Then the geometry of the model was again minimized with a RMS gradient cutoff at 0.00001 kcal·mol^−1^·Å^−1^ using the GBMV implicit model solvent with ε = 80 [44]. The obtained model were used to study the interaction energies of reagents with surrounding residues as well as to define a QM cluster used for further DFT studies of the reaction mechanism. The interaction energies were calculated using a Distance-Dependent Dielectrics model solvent (ε = 4). The same procedure was used to minimize geometry of the enzyme-product complex derived from the selected frame of MD simulation.

### 4.4. QM Modeling of the Reaction Pathway

**Figure 11:**
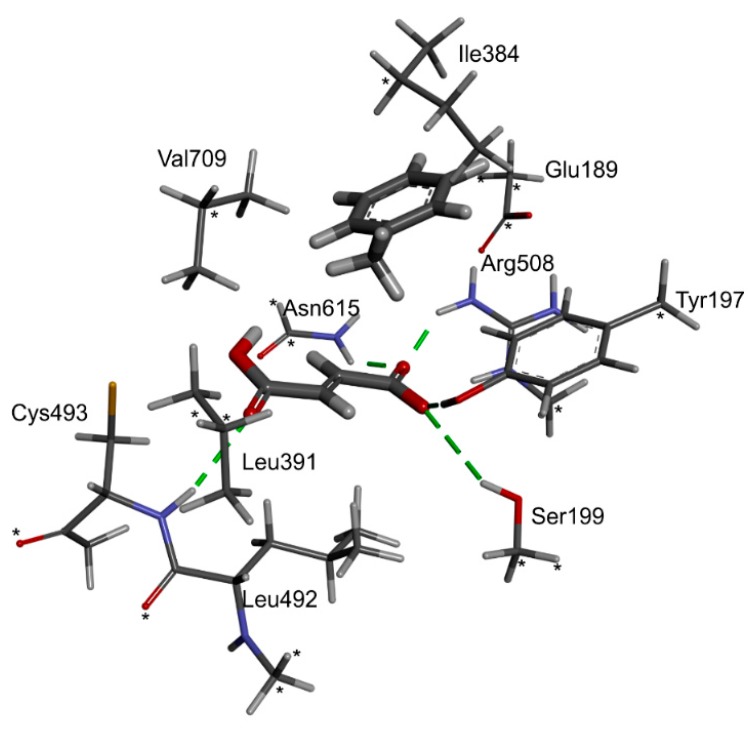
The structure of the cluster model used in QM modeling. The substrates toluene and fumarate are highlighted by thicker lines. The green lines depict hydrogen bonds stabilizing bound fumarate in the active site. Stars indicate atoms which positions were constrained in the geometry optimization.

The selected QM cluster contained whole or fragmented side chains of active site amino acids surrounding the bound substrates (*i.e.*, Glu189, Tyr197, Ser199, Ile384, Leu391, Leu492, Cys493, Arg508, and Val709—147 atoms in total) with added geometry constraints. The constraints were introduced in places where residues were cut from the main chain (and paired by H atoms) and additionally on the carbonyl and methyl capping groups of the respective side chains, mimicking the constraints set by the rest of the enzyme (see Figure 11).

Cluster model calculations were then conducted in Gaussian 09 (B3LYP functional with D2 Grimme empirical dispersion correction using a 6-31g(d,p) basis set for geometry optimization [47]). The transition states were identified by relaxed potential energy scans along selected reaction coordinates (e.g., varying C–H^…^S-Cys distances for **TS1**), followed by full optimization of the TS geometries using the Berny algorithm. Single point energies were calculated with the 6-311+g(2d,2p) basis in the gas phase, while a solvent corrections were obtained as a difference in electronic energies calculated with and without polarized continuum model (PCM) with ε = 4 at the 6-31g(d,p) level of theory [48]. The obtained electronic energies were corrected by including zero point energies (*ZPE*), thermal energy (Thermal energy), enthalpy (*H*) and Gibbs free energy (*G*) corrections obtained from B3LYP-D2/6-31G(d,p) frequency calculations (standard conditions: 1 atm., 298 K, scale factor 1) [49]. The vibration corrections were calculated with Cartesian constraints ensuring elimination of the contributions from the frozen internal coordinates to the Hessian.

### 4.5. Kinetic Isotope Effect

To calculate intrinsic kinetic isotopic effects, Gibbs free energy corrections obtained at the B3LYP/6-31g(d,p) level were calculated for toluene and d^8^-toluene for all stationary points along the reaction pathway, assuming a temperature of 298 K and pressure of 1 atm. The kinetic constants (see Table S11) were calculated according to the Equation (1):

(1) $$k_{X}=\frac{k_{B}T}{h}e^{\left(\frac{-\Delta G^{\#}}{RT}\right)}$$

where *X* stands for either H or D. The intrinsic isotope effect was calculated as the ratio of *k*_H_ to *k*_D_. The ∆*G*^#^ values were evaluated with respect to the **ES** complex for all transition states (**TS1**, **TS2** and **TS3**). Additionally, the ∆*G*^#^ value of **TS3** was also evaluated with respect to **I2**.

The experimental kinetic isotope effect for benzylsuccinate synthase was measured with [^2^H]_8_-toluene and unlabeled toluene as well as with 2,3-[^2^H]_2_-fumarate and unlabeled fumarate. The KIE was determined with a direct method in an activity assay utilizing extracts of toluene-grown *T. aromatica* cells as a catalyst and a protocol established before [14].

## 5. Conclusions

In conclusion, our studies on the active site reactivity of BSS by molecular and quantum mechanistic modeling techniques indicate that the reaction proceeds via the approach of the methyl group of toluene towards the active site thiyl radical at Cys493, generation of a benzyl radical and addition of this radical species under stereochemical inversion to the distal (C3) atom of the fumarate co-substrate bound in pro(*R*) orientation. The subsequent quenching of the product radical occurs by H-transfer from the thiol group of Cys493 to C2 of the benzylsuccinyl radical without major reorientation of the bound product. The regioselectivity of C–C bond formation is controlled by kinetic factors (combination of barriers associated with C–C bond formation and radical quenching), while the enantioselectivity of the enzyme is enforced by a combination of thermodynamic (lower probability of **ES** formation in the pro(*S*)-configuration) and kinetic factors (prohibitive barrier of the radical quenching step in the pro(*S*) pathway).

## Figures and Tables

**Table 1:** The energy profile (in kcal/mol) obtained for cluster modeling of pathway (**a**) and (**b**) (pro(*R*)). Two variants of the benzyl radical attack on the fumarate are considered (**a**) the attack on the distal carbon atom and (**b**) the attack on the proximal one (with respect to the Cys493). All energy differences were calculated with respect to **ES**.

Species	∆*E*(B3LYP+D2/6-31g(dp))	∆*E* B3LYP+D2/6-311+g(2d,2p)+ZPE + Solvent Correction	∆*E* B3LYP+D2/6-311+g(2d,2p)+Thermal Energy + Solvent Correction	∆*E* B3LYP+D2/6-311+g(2d,2p)+G + Solvent Correction
**ES**	0.00	0.00	0.00	0.00
**TS1**	20.01	14.55	13.13	18.42
**I1**	8.53	5.18	5.05	5.71
**Pathway (a)**				
**TS2a**	11.35	10.09	9.08	13.63
**I2a**	−7.28	−6.20	−7.29	−2.82
**TS3a**	14.41	10.94	9.26	15.18
**Pa**	−14.38	−11.58	−12.94	−7.71
**Pathway (b)**				
**TS2b**	14.40	11.69	10.50	15.59
**I2b**	−4.34	−4.65	−5.99	−0.12
**TS3b**	53.03	46.55	44.63	52.53
**Pb**	−8.33	−6.14	−7.98	−1.03

**Table 2:** The energy profile (in kcal/mol) obtained for cluster modeling of pathways (**c**) and (**d**) (pro(*S*)) where the benzyl radical attacks the distal carbon atom (C3) of the fumarate (with respect to the Cys493). All energy differences were calculated with respect to ES of pathway (**a**).

Species	∆*E*(B3LYP+D2/6-31g(dp))	∆*E* B3LYP+D2/6-311+g(2d,2p)+ZPE + Solvent Correction	∆*E* B3LYP+D2/6-311+g(2d,2p)+Thermal Energy + Solvent Correction	∆*E* B3LYP+D2/6-311+g(2d,2p)+G + Solvent Correction
ES pro(*R*)	0.00	0.00	0.00	0.00
**Pro(*S*)**				
ES	4.31	3.14	3.04	3.98
TS1	19.61	12.96	11.71	16.93
I1	7.79	4.14	3.78	6.42
**Pathway (c)**				
TS2c	18.93	11.17	7.95	15.93
I2c	−6.91	−6.78	−7.78	−4.86
TS3c	18.79	12.17	10.47	17.06
Pc	−14.48	−13.69	−15.26	−9.17
**Pathway (d)**				
TS2d	14.77	11.86	11.04	13.44
I2d	−4.56	−5.03	−6.09	−2.70
TS3d	42.54	31.53	30.19	34.41
Pd	−12.02	−9.57	−11.20	−5.46

## Supplementary Materials

Supplementary materials can be found at http://www.mdpi.com/1422-0067/17/4/514/s1.
