# Supplementary material for: Modeling of the Reaction Mechanism of Enzymatic Radical C–C Coupling by Benzylsuccinate Synthase
Source: Int J Mol Sci. 2016 Apr 7;17(4):514. doi: 10.3390/ijms17040514 (PMC4848970; doi:10.3390/ijms17040514)
Supplement: Supplementary file 1 [file ijms-17-00514-s001.pdf]

Supplementary Materials: Modeling of the Reaction Mechanism of Enzymatic Radical C–C Coupling by Benzylsuccinate Synthase

Maciej Szaleniec and Johann Heider

Docking and MD Study

Docking of the (*R*)- and (*S*)-Enantiomers of Benzylsuccinate (1st Stage)

In order to determine the energetically most favourable protonation state of the dicarbonic acid benzylsuccinate, all four possible protonation types of benzylsuccinate were docked into the active site. Moreover, to survey for any potential structural preferences for (*R*)-benzylsuccinate, the (*S*)-isomer was also docked into the active site. All successful docking experiments revealed that the dicarbonic acid part of the benzylsuccinates is firmly positioned between the amide backbone group of Cys493 and the guanidino group of Arg508, which had already been predicted as important amino acids of the active site [24]. The poses with the docked products were then energy-minimized *in situ* along with the surrounding residues, and the binding energies (BE) were evaluated by the "Calculate Binding Energies" protocol of DS. The BE were calculated as differences between the energy values of the protein-ligand complexes and the sum of energy values of the proteins and the respective ligands. Consequently, negative BE values indicate exergonic binding reactions, while positive values indicate endergonic binding modes that should be thermodynamically unstable. We assume here that only models yielding exergonic binding modes are plausible in the real protein.

Table S1. Binding energies (BE) to the active site in the α subunit of BSS (crystal structure) calculated for different protonation forms of both (*R*)- and (*S*)-benzylsuccinate. For most of the ligands different poses were obtained and for them the range of BE was calculated, for which the lower and upper values are provided (lowest BE ÷ highest BE). Single values are provided only if one type of the poses was obtained and was characterized by the same BE.

Type of Ligand *R*	Binding Energy (kcal/mol)	Type of Ligand *S*	Binding Energy (kcal/mol)
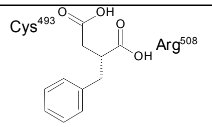	−18.7–−14.4	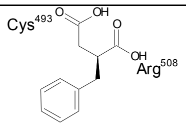	−13–−10
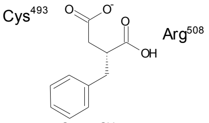	+14–+17.8	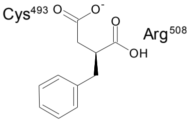	+4.0–+17.2
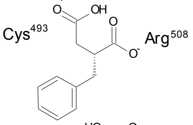	−20.8	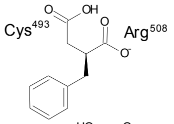	−20.8–−9.14
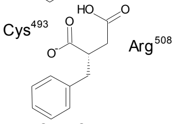	n.d.	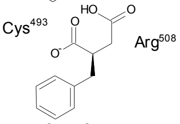	+23.1
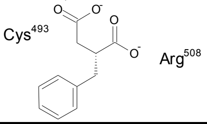	+54.6	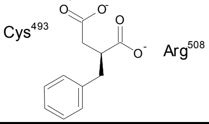	+48.7–68.4

Analysing the product binding energies obtained after geometry minimization of the active site of BSS (Table 1) revealed that the enzyme binds either completely undissociated (*R*)-benzylsuccinate or its mono-protonated form facing Arg508 with the deprotonated carboxyl group (negative BE values). All binding poses presenting a charged carboxyl group towards Cys493 showed endergonic

(positive) BE (from +14 to +17.8 kcal/mol for the mono-protonated form, 54.6 kcal/mol for the completely dissociated form). The best pose of the enzyme contained mono−protonated (*R*)-benzylsuccinate and was used in the following MD simulations.

Similar tendencies were observed for docking of (*S*)-benzylsuccinate. Although the best docking pose (mono−deprotonated oriented towards Arg508) showed the same calculated BE as for the (*R*)-isomer (−20.8 kcal/mol), we observed a higher variation on poses with that isomer, yielding more divergence of the calculated binding energies (from −20.8 to −9.14 kcal/mol), which might be due to a weaker accommodation and higher perturbation susceptibility of the product in (*S*)- than in (*R*)-configuration. However, the differences observed for the molecular interactions of BSS with the two product enantiomers were not clear enough to explain the enantioselectivity of BSS.

Docking of the Substrates and Inhibitors into the Relaxed Active Site

Table S2. Binding energies (BE) to the active site in the α subunit of BSS (model relaxed in MD simulation) calculated for toluene and different protonation forms of fumarate. For most of the ligands different poses were obtained and for them the range of BE was calculated, for which the lower and upper values are provided (lowest BE ÷ highest BE). Single values are provided only if one type of the poses was obtained and was characterized by the same BE.

Type of Ligand	Binding Energy (kcal/mol)	Type of Ligand	Binding Energy (kcal/mol)
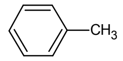	−9.9–−0.9	-	-
pro(*R*)		pro(*S*)	
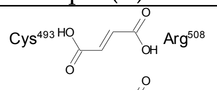	−11.7–−4.7	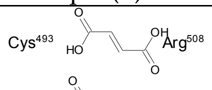	−11.5–−8.8
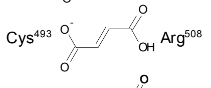	+12–+27.2	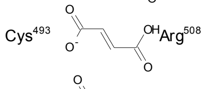	+18–+34.7
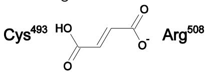	−16.7	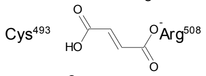	−11.2–−7
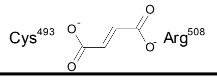	+31–+41	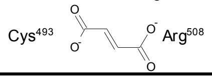	+34–+42

The structure of the BSS α subunit obtained in the initial MD simulation was used for more detailed docking studies of binding of the substrates. The results were obtained in the flexible docking protocol and were additionally assessed based on the knowledge obtained from the product docking studies (*i.e.*, orientation of the fumaric acid co-substrate between Arg508 and Cys493 of the active site).

The substrate docking studies with fumarate were consistent with the previously presented preference of the enzyme to bind preferentially the protonated or mono−deprotonated forms of the product. Exergonic binding was only predicted for mono−protonated fumarate facing Arg508 with the deprotonated group (BE −16.7 kcal/mol) or for undissociated fumarate (−12 kcal/mol). In contrast, strongly endergonic BE were predicted for monoprotonated fumarate in the inverse orientation (12–27 kcal/mol) or completely dissociated fumarate (30–40 kcal/mol). The docking studies also suggest that the enzyme does not discriminate between binding of fumarate in either pro(*R*) and pro(*S*) orientation, judging from the similar range of BE observed. Regarding the values calculated for the most favorable docking models of the mono-protonated forms, fumarate binding in the pro(*R*)-oriented mode is more favorable than in pro(*S*)-orientation by *ca.* 5 kcal/mol, but this difference is already close to the error margin of the method [38].

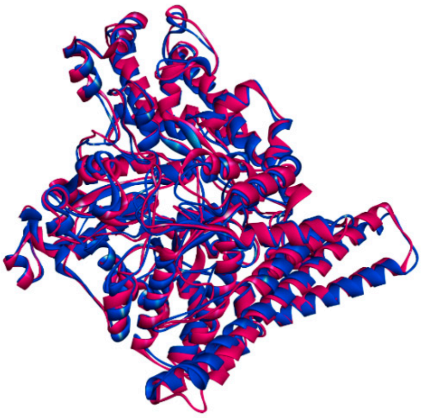

Figure S1. Comparison of the α subunit folds of the crystal structure (**blue**) and the equilibrated model of the enzyme–product complex after 5 ns MD simulation (**red**).

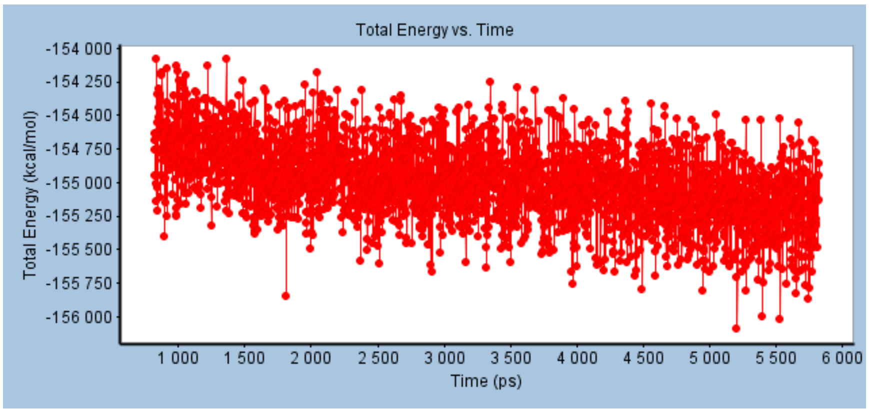

(**A**)

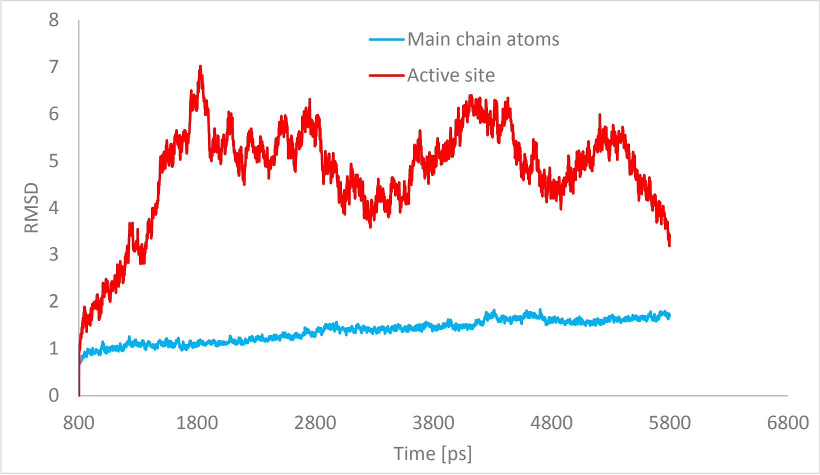

(**B**)

Figure S2. (**A**) Total energy representation of the models during the 5000 ps process of the MD production phase; (**B**) RMSD plot of for the production phase of the simulation (800–5800 ps) for main chain atoms (blue line) and all atoms of the active site including product (all residues in 5 Å radius of the product).

**Protocol Settings**	Protocol.pr_xml
**Input Receptor**	4PKF_pH7.6_dynamics_low_frame_docking.dsv
**Input Ligands**	Ligands.sd
**Input Site Sphere**	7.91601, −4.51401, 0.941172, 8
**Number of Hotspots**	100
**Docking Tolerance**	0.25
**Docking Preferences**	High Quality
**Max Hits to Save**	100
**Max Number of Hits**	100
**Minimum LibDockScore**	100
**Final Score Cutoff**	0.5
**Max BFGS Steps**	50
**Rigid Optimization**	False
**Keep Hydrogens**	False
**Max Conformation Hits**	30
**Max Start Conformations**	1000
**Steric Fraction**	0.10
**Final Cluster Radius**	0.5
**Apolar SASA Cutoff**	15.0
**Polar SASA Cutoff**	5.0
**Surface Grid Steps**	18
**Conformation Method**	CAESAR
**Maximum Conformations**	255
**Discard Existing Conformations**	True
**Energy Threshold**	20.0
**DSReport Summary**	False
**Separate Conformations**	False
**Minimization Algorithm**	Smart Minimizer
**RMSD Cutoff**	1.0
**Flexible Residues**	
**Minimization Max Steps**	1000
**Minimization RMS Gradient**	0.001
**Minimization Energy Change**	0.0
**Minimization Forcefield**	CHARMm
**Minimization Sphere of Flexible Atoms**	7.91601, −4.51401, 0.941172, 8
**Implicit Solvent Model**	Distance−Dependent Dielectrics
**Dielectric Constant**	1
**Implicit Solvent Dielectric Constant**	80
**Generalized Born Lambda Constant**	
**Minimum Hydrogen Radius**	0.8
**Use Non−polar Surface Area**	True

**Non–polar Surface Constant**	0.92
**Non–polar Surface Coefficient**	0.00542
**Salt Concentration**	0.0
**Input Atomic Radii**	van der Waals radii
**Use Molecular Surface**	True
**Nonbond List Radius**	13.0
**Nonbond Higher Cutoff Distance**	12.0
**Nonbond Lower Cutoff Distance**	9.0
**Advanced**	
**Verbose**	0
**sp2–sp2 rotation**	True
**Grid Scoring**	True
**Hotspots File**	

Interaction and Binding Energies

Table S3. Interaction energies calculated for enzyme-product complex after MD and MM minimization.

Residue	Interaction Energy (kcal/mol)	VDW Interaction Energy (kcal/mol)	Electrostatic Interaction Energy (kcal/mol)	Interaction Energy (kcal/mol)	VDW Interaction Energy (kcal/mol)	Electrostatic Interaction Energy (kcal/mol)
		**pro(*R*)**			**pro(*S*)**	
GLU189	0.76	−1.78	2.54	0.34	−2.04	2.38
PRO191	−0.25	−0.19	−0.06	−0.24	−0.19	−0.05
TYR197	−3.34	−0.18	−3.16	−3.31	−0.39	−2.92
SER199	−0.46	−0.24	−0.22	−0.49	−0.25	−0.24
SER328	−0.63	−0.41	−0.22	−1.05	−0.64	−0.41
ILE384	−2.42	−2.39	−0.03	−0.03	−0.09	0.06
PHE385	−1.09	−1.13	0.04	−2.25	−2.28	0.03
LEU391	−1.45	−1.45	−0.01	−1.22	−1.08	−0.14
LEU492	−2.56	−2.34	−0.22	−0.98	−0.87	−0.11
CYS493	−0.79	−0.54	−0.25	−1.28	−0.52	−0.76
MET494	−0.12	−0.08	−0.04	−0.09	−0.06	−0.03
SER495	−0.04	−0.20	0.16	−0.04	−0.11	0.06
ARG508	−9.53	2.40	−11.94	−11.36	−0.68	−10.67
GLY512	−0.81	−0.35	−0.47	−0.82	−0.34	−0.48
SER514	−1.35	−1.77	0.42	−1.21	−1.49	0.28
PHE516	−0.70	−0.76	0.07	−0.83	−0.82	−0.01
TRP613	−2.96	−0.43	−2.52	−0.90	−1.37	0.47
ASN615	−0.73	1.52	−2.25	−3.18	−2.32	−0.86
ILE617	−0.99	−0.90	−0.09	−0.92	−0.84	−0.08
GLN707	−3.04	−2.19	−0.85	−3.09	−2.46	−0.63
VAL709	−2.21	−1.99	−0.22	−2.89	−2.72	−0.17
LEU711	−1.89	−1.90	0.01	−3.03	−3.06	0.03
**TOTAL**	**−36.61**	**−17.29**	**−19.32**	**−38.88**	**−24.63**	**−14.25**

Table S4. Interaction energies calculated for enzyme substrates complex after MM minimization. The interaction of the enzyme is calculated together with both substrates: Fumarate and toluene.

Residue	Interaction Energy (kcal/mol)	VDW Interaction Energy (kcal/mol)	Electrostatic Interaction Energy (kcal/mol)	Interaction Energy (kcal/mol)	VDW Interaction Energy (kcal/mol)	Electrostatic Interaction Energy (kcal/mol)
		**Pro(*R*)**			**pro(*S*)**	
GLU189	0.11	−1.85	1.96	0.32	−1.63	1.95
PRO191	−0.22	−0.14	−0.08	−0.22	−0.15	−0.07
TYR197	−5.02	−2.23	−2.79	−4.24	−1.50	−2.73
SER199	−2.19	−0.55	−1.64	−2.17	−0.19	−1.98
SER328	−0.91	−0.68	−0.23	−0.95	−0.63	−0.33
ILE384	−1.89	−1.87	−0.02	−2.46	−2.44	−0.02
PHE385	−0.73	−0.73	0.00	−1.10	−1.05	−0.05
LEU391	−0.95	−0.90	−0.06	−0.64	−0.66	0.02
LEU492	−1.79	−1.18	−0.61	−2.42	−1.92	−0.50
CYS493	−3.17	−1.37	−1.80	−2.44	−1.45	−0.99
MET494	−0.24	−0.14	−0.10	−0.19	−0.11	−0.08
SER495	−0.11	−0.24	0.12	−0.01	−0.18	0.17
ARG508	−10.55	0.41	−10.96	−11.23	0.04	−11.27
GLY512	−1.19	−0.63	−0.56	−0.66	−0.38	−0.28
SER514	−1.11	−1.48	0.36	−1.15	−1.44	0.28
PHE516	−0.80	−0.78	−0.01	−0.89	−0.83	−0.06
TRP613	−1.48	−1.58	0.09	−1.56	−1.45	−0.11
ASN615	−4.04	−2.33	−1.72	−3.70	−3.09	−0.61
ILE617	−0.66	−0.74	0.08	−0.72	−0.77	0.06
GLN707	−4.53	−4.12	−0.41	−4.68	−3.44	−1.24
VAL709	−2.68	−2.40	−0.28	−2.53	−2.48	−0.05
LEU711	−2.42	−2.43	0.01	−2.40	−2.41	0.01
**TOTAL**	**−46.59**	**−27.95**	**−18.65**	**−46.03**	**−28.16**	**−17.88**

Table S5. Interaction energies calculated for enzyme substrates complex after MM minimization. The interaction is calculated between the enzyme and toluene substrate: Fumarate.

Residue	Interaction Energy (kcal/mol)	VDW Interaction Energy (kcal/mol)	Electrostatic Interaction Energy (kcal/mol)	Interaction Energy (kcal/mol)	VDW Interaction Energy (kcal/mol)	Electrostatic Interaction Energy (kcal/mol)
		**Pro(*R*)**			**pro(*S*)**	
GLU189	1.78	−0.11	1.89	1.80	−0.09	1.90
PRO191	−0.14	−0.03	−0.11	−0.14	−0.02	−0.11
TYR197	−3.45	−0.70	−2.74	−2.87	−0.19	−2.68
SER199	−2.14	−0.51	−1.64	−2.11	−0.15	−1.97
SER328	−0.83	−0.59	−0.24	−0.89	−0.55	−0.34
ILE384	−0.15	−0.11	−0.04	−0.17	−0.14	−0.04
PHE385	−0.23	−0.28	0.05	−0.43	−0.44	0.01
LEU391	−0.83	−0.82	−0.02	−0.55	−0.61	0.05
LEU492	−1.73	−1.13	−0.60	−2.36	−1.87	−0.49
CYS493	−3.09	−1.32	−1.77	−2.39	−1.41	−0.97
MET494	−0.23	−0.14	−0.09	−0.19	−0.11	−0.08
SER495	−0.11	−0.23	0.12	−0.01	−0.18	0.17
ARG508	−8.30	1.97	−10.27	−9.01	1.52	−10.54
GLY512	−1.06	−0.51	−0.55	−0.54	−0.27	−0.27
SER514	0.07	−0.14	0.21	0.05	−0.07	0.12
PHE516	−0.02	−0.01	−0.01	−0.05	−0.01	−0.04
TRP613	−1.36	−1.45	0.09	−1.46	−1.35	−0.11
ASN615	−2.22	−0.63	−1.58	−1.90	−1.39	−0.51
ILE617	0.05	−0.05	0.10	0.02	−0.05	0.07
GLN707	−3.74	−3.33	−0.40	−3.98	−2.79	−1.19
VAL709	−1.39	−1.09	−0.29	−1.39	−1.31	−0.08
LEU711	−0.06	−0.07	0.01	−0.06	−0.08	0.02
TOLUENE	−2.29	−2.64	0.34	−1.80	−2.20	0.40
**TOTAL**	**−31.46**	**−13.91**	**−17.55**	**−30.47**	**−13.77**	**−16.70**

Table S6. Interaction energies calculated for enzyme substrates complex after MM minimization. The interaction is calculated between the enzyme and fumarate and substrate: Toluene.

Residue	Interaction Energy (kcal/mol)	VDW Interaction Energy (kcal/mol)	Electrostatic Interaction Energy (kcal/mol)	Interaction Energy (kcal/mol)	VDW Interaction Energy (kcal/mol)	Electrostatic Interaction Energy (kcal/mol)
		**Pro(*R*)**			**pro(*S*)**	
GLU189	−1.67	−1.75	0.08	−1.48	−1.53	0.05
PRO191	−0.08	−0.12	0.03	−0.08	−0.13	0.04
TYR197	−1.57	−1.52	−0.05	−1.37	−1.32	−0.05
SER199	−0.05	−0.05	0.00	−0.06	−0.04	−0.02
SER328	−0.08	−0.09	0.01	−0.06	−0.08	0.01
ILE384	−1.74	−1.76	0.02	−2.28	−2.30	0.02
PHE385	−0.50	−0.45	−0.05	−0.66	−0.61	−0.05
LEU391	−0.12	−0.08	−0.04	−0.09	−0.05	−0.03
LEU492	−0.06	−0.05	−0.01	−0.06	−0.04	−0.01
CYS493	−0.08	−0.05	−0.04	−0.05	−0.03	−0.01
MET494	−0.01	0.00	−0.01	0.00	0.00	0.01
SER495	0.00	−0.01	0.01	0.00	−0.01	0.01
ARG508	−2.26	−1.56	−0.69	−2.22	−1.48	−0.74
GLY512	−0.14	−0.12	−0.01	−0.12	−0.11	0.00
SER514	−1.19	−1.34	0.16	−1.20	−1.36	0.16
PHE516	−0.77	−0.77	0.00	−0.84	−0.82	−0.01
TRP613	−0.12	−0.12	0.00	−0.10	−0.10	−0.01
ASN615	−1.82	−1.69	−0.13	−1.80	−1.70	−0.10
ILE617	−0.71	−0.69	−0.02	−0.73	−0.72	−0.01
GLN707	−0.79	−0.78	−0.01	−0.70	−0.65	−0.04
VAL709	−1.29	−1.31	0.02	−1.14	−1.17	0.03
LEU711	−2.37	−2.36	−0.01	−2.33	−2.33	−0.01
Fumaric acid	−2.29	−2.64	0.34	−1.80	−2.20	0.40
**TOTAL**	**−19.72**	**−19.31**	**−0.41**	**−19.17**	**−18.80**	**−0.37**

Table S7. Results of binding energy calculations for BSS-product complexes. All energies in kcal/mol.

benzylsuccinate	Binding Energy	Total Binding Energy	Ligand Energy	Protein Energy	Complex Energy	Entropic Energy	Ligand Conformational Energy	Ligand Conformational Entropy [kcal/mol K]
R	−113.59	−112.77	−99.27	−51976.160	−52189.01	18.45	0.82	0.82127
S	−106.56	−103.93	−108.50	−51984.43	−52199.49	18.37	2.63	0.62

Table S8. Results of binding energy calculations for BSS-substrates complexes. All energies in kcal/mol.

Toluene and Fumarate	Binding Energy	Total Binding Energy	Ligand Energy	Protein Energy	Complex Energy	Entropic Energy	Ligand Conformational Energy	Ligand Conformational Entropy [kcal/mol K]
Pro(*R*)	−132.7234	−131.90	−114.8967	−52056.4062	−52304.0262	18.5583	0.82127	0.82127
Pro(*S*)	−123.4308	−122.61	−112.6843	−52031.2708	−52267.3859	18.5840	0.82127	0.82127

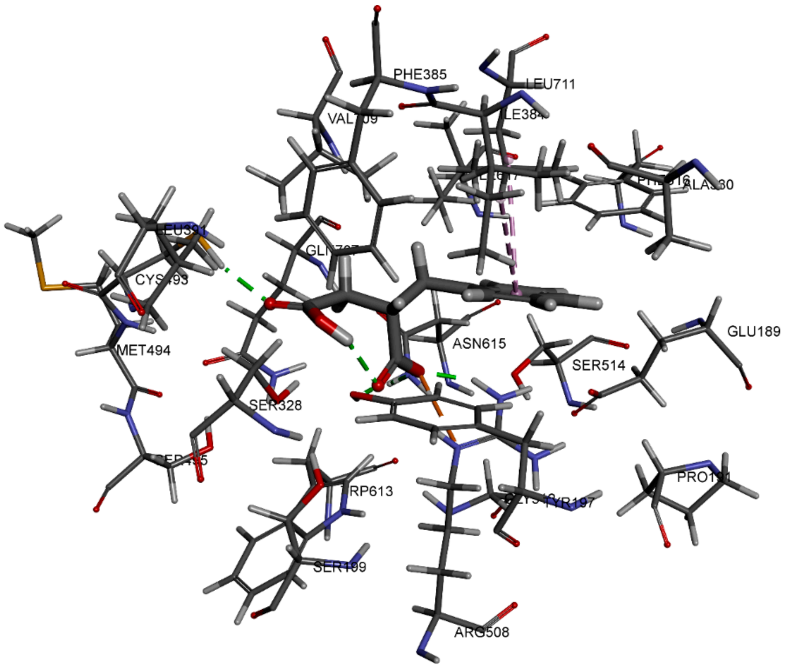

Figure S3. Structural model of the enzyme-product complex. Binding mode of the (*S*)-benzylsuccinate (monoprotonated).

QM Modelling Results

Figures Presenting Stationary Points of Pathway a

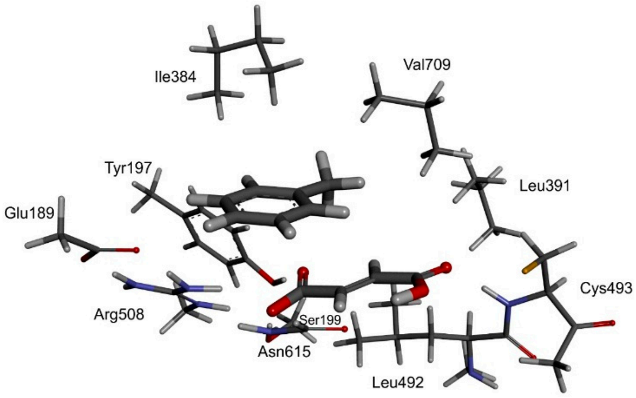

Figure S4. ES of the pathway a.

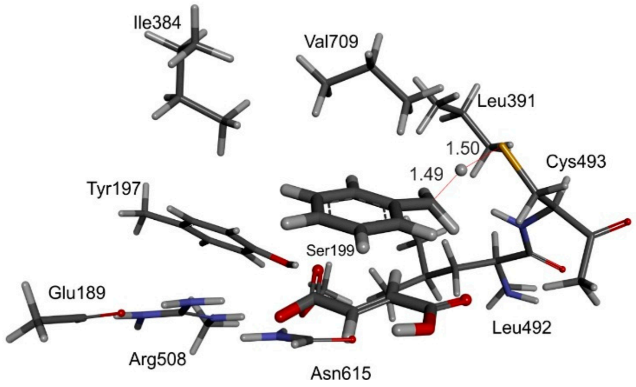

Figure S5. TS1 of the pathway a.

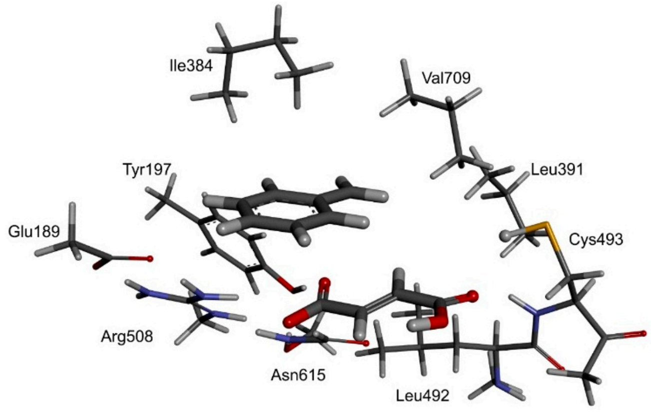

Figure S6. I1 of the pathway a.

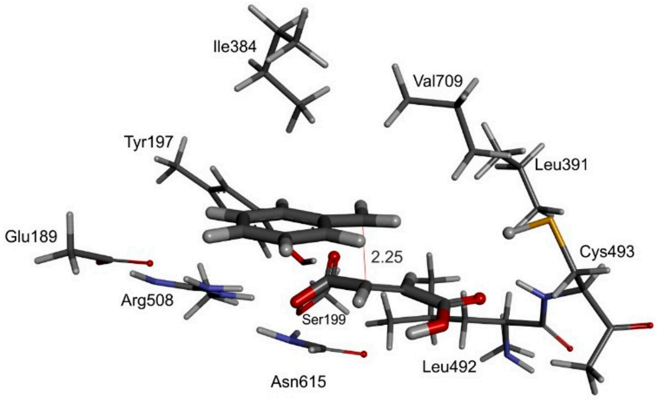

Figure S7. TS2a of the pathway a.

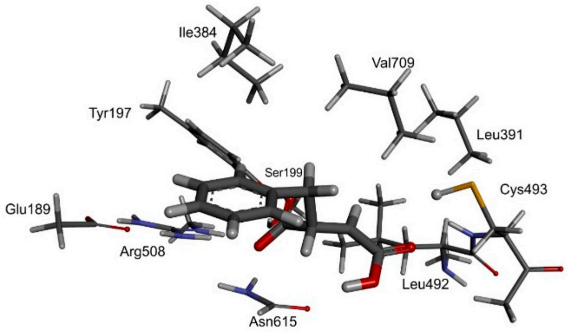

Figure S8. I2a of the pathway a.

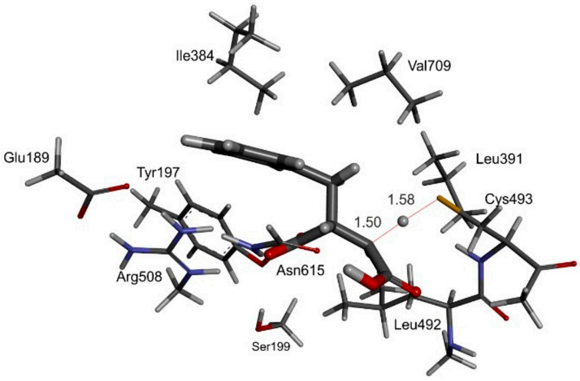

Figure S9. TS3a of the pathway a.

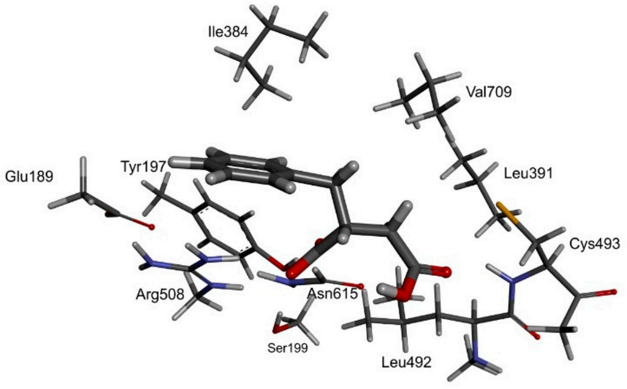

Figure S10. Pa of the pathway a.

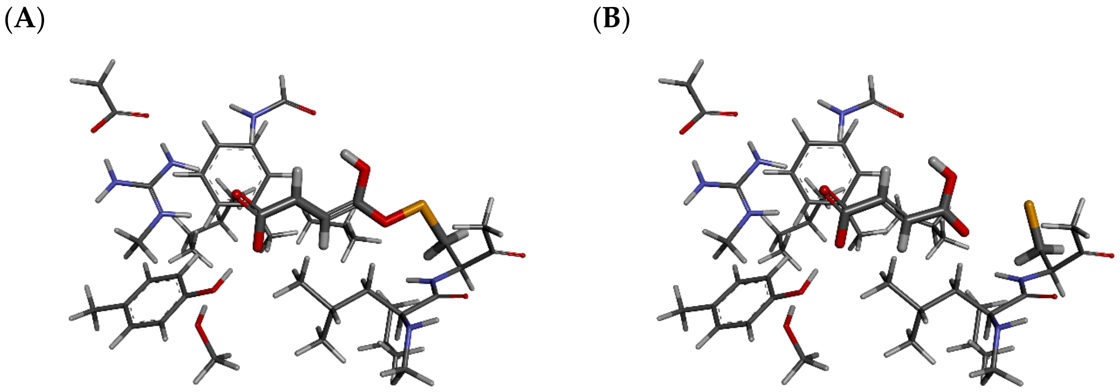

Figure S11. The initial steps of the pathway d. (**A**) Intermediate with the S–O bond length constrained; (**B**) the structure obtained after the constrains on d(S–O) is removed.

Energies Obtained in QM Modeling

Table S9. Energies and vibrational corrections [a.u.] calculated for pathway (a) and (b) (pro (*R*)).

Stationary Point	*E* B3LYP+ D2/6-31g(dp)	*E* PCM at B3LYP+ D2/6-31g(dp)	*E* B3LYP+D2/6-311 +g(2d,2p)+	*ZPE* Correction	Thermal Energy Correction	*H* Correction	*G* Correction
**ES**	−3320.0908451	−3320.1394802	−3320.9857151	1.162609	1.228058	1.229003	1.056450
**ES***	−3320.0842464	−3320.1336787	−3320.9806983	1.165435	1.229469	1.230413	1.064202
**TS1**	−3320.0589624	−3320.1100088	−3320.9575363	1.160022	1.223219	1.224164	1.060029
**I1**	−3320.0772589	−3320.1251951	−3320.9755448	1.159999	1.225239	1.226183	1.054674
			**Pathway (a)**				
**TS2a**	−3320.0727530	−3320.1199040	−3320.9696369	1.161134	1.224965	1.225909	1.060609
**I2a**	−3320.1024480	−3320.1498662	−3320.9979840	1.163774	1.227499	1.228443	1.063010
**TS3a**	−3320.0678806	−3320.1180934	−3320.9628245	1.158736	1.221507	1.222452	1.059326
**Pa**	−3320.1137596	−3320.1632702	−3321.0071811	1.166500	1.229779	1.230723	1.066498
			**Pathway (b)**				
**TS2b**	−3320.0678894	−3320.1173531	−3320.9649943	1.161345	1.224894	1.225838	1.061395
**I2b**	−3320.0977675	−3320.1482853	−3320.9927426	1.164102	1.227421	1.228366	1.065166
**TS3b**	−3320.0063315	−3320.0596865	−3320.9040344	1.159825	1.222221	1.223165	1.063208
**Pb**	−3320.1041223	−3320.1568537	−3320.9966539	1.167855	1.230371	1.231315	1.069839

ES*—ES complex with toluene position as in the crystal structure; geometry calculated with additional S–C constrain.

Table S10. Energies and vibrational corrections [a.u.] calculated for pathway (c) and (d) (pro *S*).

Stationary Point	*E* B3LYP+D2/6-31g(dp)	*E* PCM at B3LYP+ D2/6-31g(dp)	*E* B3LYP+D2/6-311+ g(2d,2p)+	*ZPE* Correction	Thermal Energy Correction	*H* Correction	*G* Correction
**ES**	−3320.083978	−3320.133712	−3320.979786	1.162762	1.228054	1.228998	1.057935
**ES***	−3320.079548	−3320.129428	−3320.977458	1.164326	1.228872	1.229816	1.061106
**TS1**	−3320.059587	−3320.110891	−3320.958972	1.159193	1.222642	1.223586	1.059363
**I1**	−3320.078431	−3320.126387	−3320.977776	1.258976	1.331928	1.332872	1.135721
			**Pathway (c)**				
**TS2c**	−3320.060679	−3320.109139	−3320.962375	1.156901	1.217206	1.218151	1.058316
**I2c**	−3320.101862	−3320.149904	−3320.997733	1.163224	1.227089	1.228033	1.060122
**TS3c**	−3320.060894	−3320.11307	−3320.958494	1.158321	1.221066	1.222010	1.059960
**Pc**	−3320.113918	−3320.165038	−3321.009834	1.167395	1.230349	1.231293	1.068440
			**Pathway (d)**				
**TS2 d**	−3320.067311	−3320.115798	−3320.964667	1.160309	1.224451	1.225395	1.056667
**I2 d**	−3320.098116	−3320.147235	−3320.994102	1.163469	1.227221	1.228165	1.061013
**TS3 d**	−3320.023049	−3320.076091	−3320.926227	1.157780	1.221095	1.222039	1.056205
**Pd**	−3320.109993	−3320.159980	−3321.004855	1.167848	1.230696	1.231640	1.068242

Table S11. The vibrational corrections (in a.u.) and the energy profile (in kcal/mol) obtained for cluster modelling pathways **a** and **b** and [^{2}H]$_8$-toluene as a substrate. Corrections were obtained for standard conditions (298.15 K, 1 atm., scale factor = 1).

	ZPE Corrections	Thermal Energy Corrections	*H* Corrections	*G* Corrections	Δ*E* B3LYP+D2/6-311+g(2d,2p)+ZPE + Solvent Corr.	Δ*E* B3LYP+D2/6-311 +g(2d,2p)+Thermal Energy + Solvent Corr.	Δ*E* B3LYP+D2/6-311+g(2d,2p)+H and Solvent Corr.	Δ*E* B3LYP+D2/6-311 +g(2d,2p)+G and Solvent Corr.
				**Pathway a with toluene**				
**ES**	1.162609	1.228058	1.229003	1.056450	0.00	0.00	0.00	0.00
**TS1**	1.165435	1.229469	1.230413	1.064202	14.55	13.13	13.13	18.42
**I1**	1.160022	1.223219	1.224164	1.060029	5.18	5.05	5.05	5.71
**TS2a**	1.161134	1.224965	1.225909	1.060609	10.09	9.08	9.08	13.63
**I2a**	1.163774	1.227499	1.228443	1.063010	−6.20	−7.29	−7.29	−2.82
**TS3a**	1.158736	1.221507	1.222452	1.059326	10.94	9.26	9.26	15.18
**Pa**	1.166500	1.229779	1.230723	1.066498	−11.58	−12.94	−12.94	−7.71
				**Pathway a with [^{2}H]$_8$-toluene**				
**ES**	1.136504	1.202958	1.203902	1.029422	0.00	0.00	0.00	0.00
**TS1**	1.135583	1.199914	1.200858	1.034825	15.59	14.26	14.26	19.56
**I1**	1.13496	1.201486	1.202431	1.028539	5.85	5.90	5.90	6.27
**TS2a**	1.135703	1.200733	1.201677	1.034291	10.52	9.62	9.62	14.08
**I2a**	1.138076	1.202922	1.203866	1.036469	−5.95	−6.96	−6.96	−2.51
**TS3a**	1.134227	1.198063	1.199006	1.034097	11.95	10.30	10.30	16.31
**Pa**	1.139999	1.204373	1.205317	1.039182	−11.83	−13.13	−13.13	−7.89
		**Rate for toluene [s^{-1}]**				**Rate for and [^{2}H]$_8$-toluene [s^{-1}]**		
	**Δ*ZPE*$^{\#}$**	**ΔThem. Energy $^{\#}$**	**Δ*H*$^{\#}$**	**Δ*G*$^{\#}$**	**Δ*ZPE*$^{\#}$**	**ΔThem. Energy $^{\#}$**	**Δ*H* $^{\#}$**	**Δ*G* $^{\#}$**
**TS1**	2.15×10^{-11}	2.34×10^{-10}	2.34×10^{-10}	3.12×10^{-14}	3.68×10^{-12}	3.49×10^{-11}	3.49×10^{-11}	4.52×10^{-15}
**TS2a**	3.95×10^{-8}	2.19×10^{-7}	2.20×10^{-7}	1.01×10^{-10}	1.93×10^{-8}	8.74×10^{-8}	8.74×10^{-8}	4.75 × 10−11
**TS3a**	9.42×10^{-9}	1.61×10^{-7}	1.61 × 10−7	7.38×10^{-12}	1.74×10^{-9}	2.78×10^{-8}	2.79×10^{-8}	1.10×10^{-12}
**TS3a *vs.* I2**	2.66×10^{-13}	7.30×10^{-13}	7.29×10^{-13}	6.32×10^{-14}	7.53×10^{-14}	2.20×10^{-13}	2.20×10^{-13}	1.57×10^{-14}
							k_H/k_D	
		**Transition State**			**Δ*ZPE*$^{\#}$**	**ΔThem. Energy $^{\#}$**	**Δ*H* $^{\#}$**	**Δ*G* $^{\#}$**
		**TS1**			5.844	6.700	6.700	6.909
		**TS2a**			2.043	2.509	2.511	2.122
		**TS3a**			5.426	5.782	5.776	6.728
		**TS3a *vs.* I2**			3.525	3.322	3.315	4.016
